# Metabolic plasticity maintains proliferation in pyruvate dehydrogenase deficient cells

**DOI:** 10.1186/s40170-015-0134-4

**Published:** 2015-06-29

**Authors:** Kartik N. Rajagopalan, Robert A. Egnatchik, Maria A. Calvaruso, Ajla T. Wasti, Mahesh S. Padanad, Lindsey K. Boroughs, Bookyung Ko, Christopher T. Hensley, Melih Acar, Zeping Hu, Lei Jiang, Juan M. Pascual, Pier Paolo Scaglioni, Ralph J. DeBerardinis

**Affiliations:** Children’s Medical Center Research Institute, University of Texas Southwestern Medical Center, Dallas, TX 75390-8502 USA; Department of Internal Medicine, University of Texas Southwestern Medical Center, Dallas, TX 75390-8502 USA; Departments of Neurology, University of Texas Southwestern Medical Center, Dallas, TX 75390-8502 USA; Departments of Pediatrics, University of Texas Southwestern Medical Center, Dallas, TX 75390-8502 USA; McDermott Center for Human Growth and Development, University of Texas Southwestern Medical Center, Dallas, TX 75390-8502 USA

## Abstract

**Background:**

Pyruvate dehydrogenase (PDH) occupies a central node of intermediary metabolism, converting pyruvate to acetyl-CoA, thus committing carbon derived from glucose to an aerobic fate rather than an anaerobic one. Rapidly proliferating tissues, including human tumors, use PDH to generate energy and macromolecular precursors. However, evidence supports the benefits of constraining maximal PDH activity under certain contexts, including hypoxia and oncogene-induced cell growth. Although PDH is one of the most widely studied enzyme complexes in mammals, its requirement for cell growth is unknown. In this study, we directly addressed whether PDH is required for mammalian cells to proliferate.

**Results:**

We genetically suppressed expression of the *PDHA1* gene encoding an essential subunit of the PDH complex and characterized the effects on intermediary metabolism and cell proliferation using a combination of stable isotope tracing and growth assays. Surprisingly, rapidly dividing cells tolerated loss of PDH activity without major effects on proliferative rates in complete medium. PDH suppression increased reliance on extracellular lipids, and in some cell lines, reducing lipid availability uncovered a modest growth defect that could be completely reversed by providing exogenous-free fatty acids. PDH suppression also shifted the source of lipogenic acetyl-CoA from glucose to glutamine, and this compensatory pathway required a net reductive isocitrate dehydrogenase (IDH) flux to produce a source of glutamine-derived acetyl-CoA for fatty acids. By deleting the cytosolic isoform of IDH (IDH1), the enhanced contribution of glutamine to the lipogenic acetyl-CoA pool during *PDHA1* suppression was eliminated, and growth was modestly suppressed.

**Conclusions:**

Although PDH suppression substantially alters central carbon metabolism, the data indicate that rapid cell proliferation occurs independently of PDH activity. Our findings reveal that this central enzyme is essentially dispensable for growth and proliferation of both primary cells and established cell lines. We also identify the compensatory mechanisms that are activated under PDH deficiency, namely scavenging of extracellular lipids and lipogenic acetyl-CoA production from reductive glutamine metabolism through IDH1.

**Electronic supplementary material:**

The online version of this article (doi:10.1186/s40170-015-0134-4) contains supplementary material, which is available to authorized users.

## Background

The importance of metabolism in cell growth and proliferation is illustrated by its emerging role as a molecular hallmark and source of therapeutic targets in cancer [1] and the intimate connection between oncogenic mutations and metabolic reprogramming [2]. Observations made by Otto Warburg documented enhanced glucose uptake and increased lactate secretion in cancer cells relative to differentiated tissues [3]. Specifically, cancer cells were found to convert a high fraction of glucose-derived carbon to lactate rather than oxidizing it to CO_2_ in the mitochondria. This phenomenon has been called aerobic glycolysis (or, more commonly, the Warburg effect) because it occurs even when enough oxygen is present to support normal mitochondrial function. Evidence indicates that aerobic glycolysis supports cell survival and growth in numerous ways, including providing substrate for macromolecular synthesis [4, 5], apoptosis resistance [6, 7], and evasion of senescence during oncogenic transformation [8, 9].

However, many of the biosynthetic activities of proliferating cells involve mitochondrial metabolism. For example, the TCA cycle generates precursors to synthesize proteins, nucleic acids, and lipids, as well as providing reducing equivalents to drive electron-transport chain flux and oxidative phosphorylation [10, 11]. The pyruvate dehydrogenase complex (PDH) occupies a crucial node in glucose metabolism, as it oxidatively decarboxylates pyruvate generated from glycolysis or other pathways to generate acetyl-CoA for the TCA cycle, thus separating pyruvate between aerobic and anaerobic metabolism. The complex functions as a series of three distinct enzymes to produce acetyl-CoA from pyruvate, including pyruvate dehydrogenase, dihydrolipoamide acetyltransferase, and dihydrolipoamide dehydrogenase, catalyzed by the E1, E2, and E3 enzymes, respectively. Pyruvate decarboxylation catalyzed by E1 is considered to be the rate-limiting step. E1 is composed of two α and two β subunits, with the E1α subunit encoded by the *PDHA1* gene [12]. *PDHA1* resides on the X chromosome in both humans and mice, and human males hemizygous for loss-of-function *PDHA1* mutations display severe lactic acidosis [13]. The activity of PDH is subject to many levels of regulation, including calcium concentration, energy status, substrate availability, the NAD+/NADH ratio, and post-translational modifications, particularly inhibitory serine phosphorylation of E1α by pyruvate dehydrogenase kinases (PDKs) [14].

PDH’s requirement for cell growth is incompletely characterized and appears to be complex. On the one hand, PDK-dependent suppression of maximal PDH activity has been demonstrated to occur in normoxic cancer cells, and PDK1 has been linked to the growth-promoting benefits of aerobic glycolysis in culture and in vivo [15, 16]. Stimulation of PDH activity through pharmacological PDK inhibition has shown therapeutic promise in preclinical and early clinical studies [7, 17]. On the other hand, glucose-dependent fatty-acid synthesis and other growth-promoting biosynthetic pathways require PDH, and cultured cancer cells typically display activity of the enzyme [18, 19]. Perhaps more importantly, the few studies that have probed metabolic activity in human tumors have demonstrated clear evidence of PDH activity in vivo [20–23]. These data could be interpreted to indicate that PDH activity is maintained within a fairly narrow range in proliferating cells, with a modest level needed to support energy and macromolecule formation, while avoiding excess activity reduces oxidative stress and other problems associated with dysregulated oxidative metabolism.

Although extensive data have indicated the importance of constraining maximal PDH activity, the need for basal levels of PDH activity to support cell metabolism and growth in proliferating cells has not been carefully studied. Here we silenced or deleted the E1α subunit of PDH in cultured cells. Surprisingly, dividing cells tolerated loss of PDH activity—including genetic ablation of *PDHA1*—without major effects on their proliferative rate in complete medium. Scavenging lipids from the medium and converting to a glutamine-dependent form of fatty-acid synthesis were both activated during PDH suppression.

## Methods

### Cell lines

shRNA hairpins were generated using the Hemann Lab shRNA database and cloned into the tetracycline-inducible plasmid TtRMPVIR (Addgene plasmid 27995) generated by the Lowe Lab [24] using the In-Fusion HD cloning kit (Clontech). Sequences of control (shNS) and two hairpins against *PDHA1* (shPDHA1-1 and shPDHA1-2) are contained in Additional file 1: Table S1. Retroviruses were generated in 293FT cells. H460 and SFXL cells were infected with shRNA-expressing retroviruses. Then, infected pools were sorted by FACS for the highest 10 % of YFP expression (constitutive marker). shRNA expression was induced with 100 ng/mL doxycycline (Research Products International) for 4 days. IDH1 knockout H460 cell lines were generated using CRISPR/Cas9 [25]. Wild-type clones were selected from both control and targeting vector transfections. To control for variations among individual clones, four to five clones were pooled, and different pools for each targeted gene were used for further experiments. *PDHA1* knockout MEFs were created by breeding *PDHA1FLex8/Y* with *UBC-Cre-ER*; *X/X* mice to generate a litter that contained *UBC-Cre-ER*; *X/Y* and *PDHA1FLex8/X*. These mice were then interbred, and fibroblasts were isolated from one embryo with the genotype *UBC-Cre-ER*; *PDHA1FLex8/Y. PDHA1* was deleted in vitro using 500 nM 4-hydroxytamoxifen. Control (*PDHA1FLex8/Y*) and *PDHA1*-deficient (*PDHA1Δex8/Y*) single clones were isolated by FACS. All mouse studies followed international guidelines for the care and experimental use of animals. These studies were performed under a protocol (2007–0223) approved by the UT Southwestern Institutional Animal Care and Use Committee (IACUC).

### Cell culture and growth assays

H460 and SFXL cells were cultured in D5796 Dulbecco’s Modified Eagle Medium (DMEM) supplemented with 5 % tetracycline-reduced fetal bovine serum (FBS) (Fisher Scientific), 2 mM glutamine, and penicillin/streptomycin. MEFs were cultured in identical conditions except that normal FBS was used (Hyclone). Cells were detached from the plate for passaging using 0.05 % trypsin (Hyclone). For growth curves, 1×10^5^ cells were plated and allowed to proliferate (4 days for SFXL and H460; 2 days for MEFs). Cells were counted using a Vi-Cell XR Cell Viability Analyzer (Beckman-Coulter).

### Western blotting

Protein was extracted from cells using RIPA buffer with protease inhibitors (Roche). Protein concentration was measured using the BCA protein assay (ThermoScientific). Protein was separated by SDS/PAGE and transferred to a PVDF membrane, which was probed with antibodies against PDHE1α (Invitrogen), IDH1 (Abcam), or cyclophilin B (Abcam). Secondary antibodies were anti-mouse or anti-rabbit (both Jackson ImmunoResearch).

### PDH activity measurements

PDH activity was measured as described [26] with modifications. Briefly, cells were incubated for 1 h at 37 °C with 5 mM sodium dichloroacetate (DCA) to activate PDH. After incubation, cells were trypsinized, counted, and 30–40×10^6^ cells were re-suspended in 6 mL of cold isolation buffer and homogenized in a tight-fitting ground-glass Potter. Mitochondrial pellets were isolated by centrifugation and stored at −80 °C. For the assay, the pellet was re-suspended and pre-incubated in buffer containing DCA for 10 min at 37 °C. An aliquot was used for protein quantification, and the PDH activity assay was performed at 25 °C in a 96-well plate with each well containing 100 μg of mitochondrial suspension and reaction mixture. PDH activity was determined by following reduction of the MTT dye. The μmoles of pyruvate oxidized were calculated from the absorbance change in MTT at 570 nm [26]. Non-specific dye reduction was measured in the presence of 5 mM 3-bromopyruvate and subtracted to achieve the final PDH activity value.

### Metabolic assays and stable isotope tracing

Culture media was collected at the end of the incubation and analyzed for glucose, lactate, glutamine, and glutamate content using an automated electrochemical analyzer (NOVA BioProfile Basic-4 Analyzer). Aspartate and alanine were measured using high-pressure liquid chromatography (Hitachi L8900). Net consumption and secretion rates were normalized to cell protein content. For ^13^C tracing, when cells reached 50 % confluence, they were labeled with the tracer of interest, either 10-mM [U-^13^C]glucose or 4-mM [U-^13^C]glutamine, in DMEM supplemented with 5 % dialyzed FBS and allowed to proliferate for 24 h. Metabolites were extracted using the Bligh-Dyer method [27]. Assessment of metabolite enrichment was performed as previously described [28, 29]. Metabolite levels were determined by normalizing to an internal standard (sodium 2-oxobutyrate) and protein content. For acetyl-carnitine measurements, dishes of 80–90 % confluent cells were incubated in 10-mM glucose and 4-mM glutamine for 6 h. Extraction of metabolites, preparation for profiling by LC/MS/MS, and data analysis were performed as described [30]. Metabolite abundance was normalized to protein content.

### Radiolabeling of lipids

Cells were incubated with [U-^14^C]glucose (140 μCi, 1 μM) or [U-^14^C]glutamine (25 μCi, 180 nM) in DMEM supplemented with 5 % tetracycline-reduced FBS and allowed to proliferate for 24 h, then lipids were extracted [27]. Lipids were separated using a silica gel glass-backed plate (Fisher) in polar and non-polar solvents alongside TLC reference standards 18–5 and 18–6 (Nu-Chek Prep). Lipid bands were developed in iodine, removed from glass plates, and placed in Ecolume Liquid Scintillation Cocktail (MP Biomedicals). Counts per minute (CPM) were determined through scintillation. CPM signal was normalized to protein content for each sample. The abundance of ^14^C in lipids was calculated using a standard curve.

### BODIPY FA labeling of H460 cells

H460 cells were cultured with Bodipy 500/510 C4-C9 (Molecular Probes) in DMEM containing delipidated serum for 24 h. Cells were fixed with 4 % formaldehyde in phosphate-buffered saline (PBS) for 30 min, washed three times in PBS, and stained for Hoechst for 10 min in PBS. The cells were mounted on a slide with Fluoromount-G (Southern Biotech). Images were acquired with a LEICA DM5500B fluorescent microscope equipped with LEICA DFC340FX digital camera at ×20 magnification. ImageJ software was used to quantify signal intensity for BODIPY and DAPI (used for normalization) to determine extent of fatty-acid uptake.

### Serum delipidation and fatty-acid rescue

Delipidated fetal calf serum was prepared as described [31]. Briefly, lipids were extracted by mixing serum with n-butanol and isopropyl ether at room temperature followed by incubation on ice. The aqueous layer was extracted by centrifugation, serially re-extracted in isopropyl ether, and evaporated under nitrogen gas. The lyophilate was dissolved in distilled water and dialyzed against phosphate-buffered saline (PBS). The average concentrations of various lipid species before and after delipidation have been measured [32]. For fatty-acid rescue, palmitate and oleate were dissolved in ethanol and complexed to fatty acid-free bovine serum albumin (Sigma) dissolved in PBS. The fatty-acid mixture was directly added to DMEM containing delipidated serum to make fatty-acid rescue medium.

### Metabolic flux analysis (MFA)

Steady state metabolic fluxes were calculated by combining extracellular flux rates, growth rates, and ^13^C mass isotopomer distributions (MIDs) using the INCA software package [33], which applies an elementary metabolite unit framework to efficiently simulate MIDs [34, 35]. We developed a reaction network describing the stoichiometry and carbon transitions involved in glycolysis, the TCA cycle, and biomass generation. Parallel labeling data from cells fed either [U-^13^C] glucose or [U-^13^C]glutamine were used to simultaneously fit the same network model to estimate intracellular fluxes. To ensure that a global minimum of fluxes was identified, flux estimations were initiated from random values and repeated a minimum of 50 times. A chi-square test was applied to test goodness-of-fit, and accurate 95 % confidence intervals were calculated by assessing the sensitivity of the sum of squared residuals to flux parameter variations [36].

## Results

### Metabolic effects of *PDHA1* silencing

**Fig. 1:**
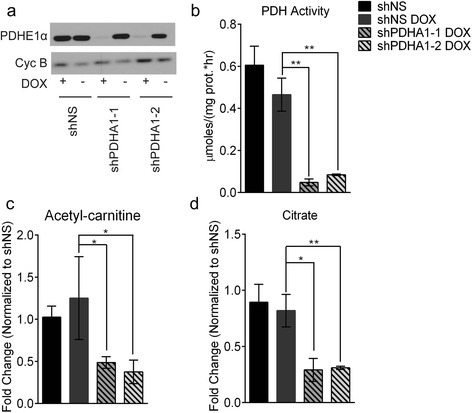
Suppression of PDH E1α reduces abundance of metabolites downstream of PDH. **a** Western blot analysis for PDH E1α. Cyclophilin B was used as a loading control. **b** PDH activity from control cells and cells expressing *PDHA1* shRNAs. **c**, **d** Intracellular levels of acetyl-carnitine and citrate. Values are the average and SD of biological triplicates, except (**b**) which represents technical triplicates. *P < 0.05; **P < 0.005

PDH activity was suppressed in H460 non-small cell lung cancer cells using doxycycline-inducible shRNAs directed against the *PDHA1* transcript. Induction of either of two hairpins greatly reduced PDH E1α protein levels (Fig. 1a) and PDH enzyme activity (Fig. 1b). *PDHA1* suppression did not alter glucose consumption or lactate secretion (Additional file 1: Figure S1a) but caused a small increase in glutamine consumption and a decrease in glutamate secretion (Additional file 1: Figure: S1b) and reduced intracellular abundance of acetyl-carnitine (Fig. 1c) and citrate (Fig. 1d), two metabolites generated from acetyl-CoA. Levels of intracellular and extracellular aspartate and alanine were enhanced during *PDHA1* silencing (Additional file 1: Figure S1c–f), possibly reflecting excess OAA and pyruvate, respectively [29, 37, 38].

**Fig. 2:**
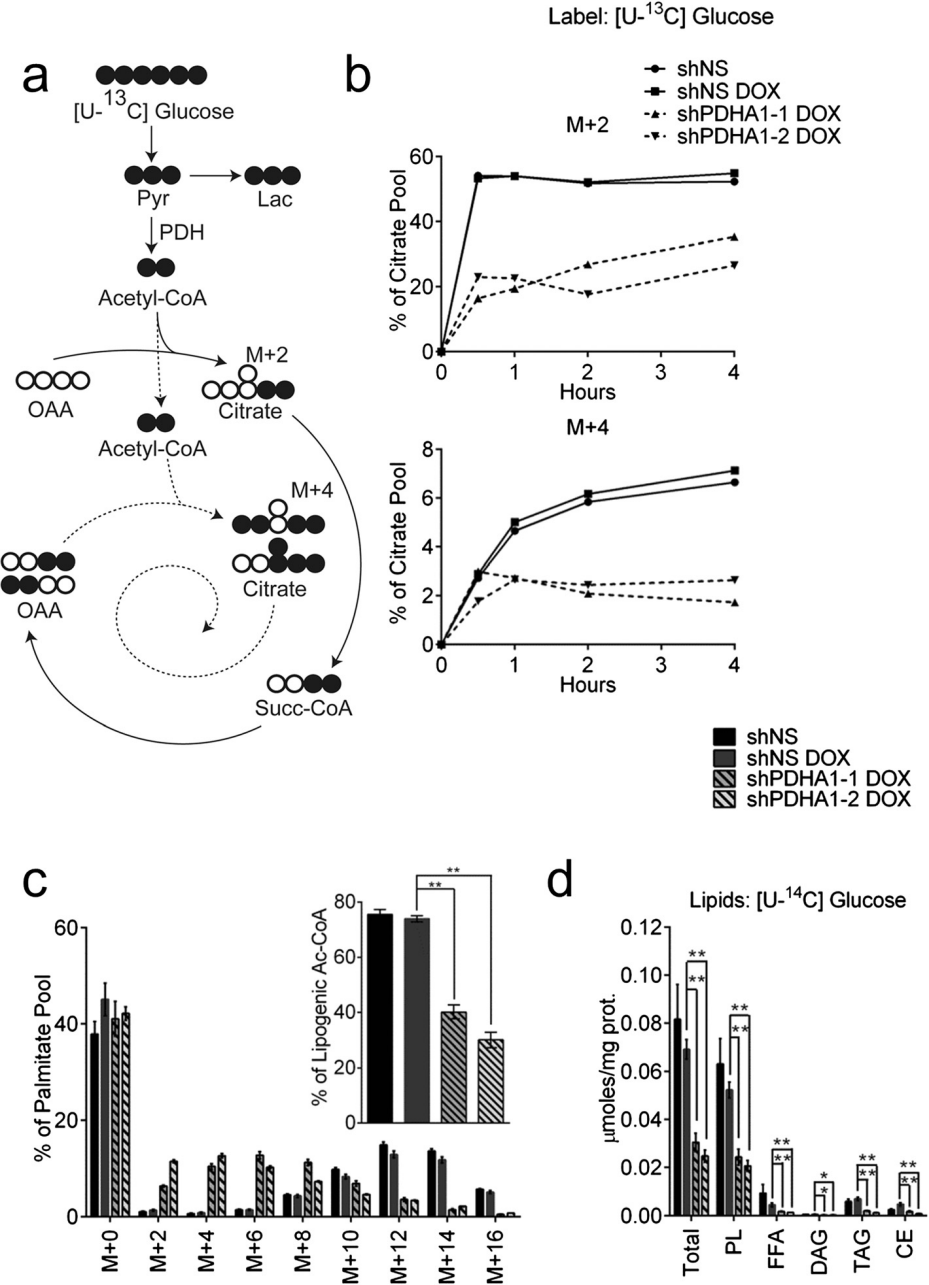
Suppression of PDH activity reduces transfer of glucose carbon into citrate and lipids. **a** Schematic representing incorporation of ^13^C derived from glucose into metabolites of glycolysis and the TCA cycle. *White and black circles* are ^12^C and ^13^C, respectively. The *solid line* represents the first turn of the TCA cycle, and the *dotted line* represents the second turn. **b** Fractional abundance of citrate m + 2 and m + 4 during culture of H460 cells with [U-^13^C]glucose. **c** Fractional abundance of palmitate isotopologues after 24 h of culture in [U-^13^C]glucose. The *inset* shows the percentage of lipogenic acetyl-CoA derived from glucose carbon. **d** Labeling of lipid species from [U-^14^C]glucose. Data are the average and SD of biological triplicates, except for the *inset* in (**c**), where *error bars* represent 95 % CI. *P < 0.05; **P < 0.005. *Abbreviations*: *CE* cholesteryl esters, *DAG* diacylglycerol, *FFA* free fatty acids, *Lac* lactate, *OAA* oxaloacetate, *PL* phospholipids, *Pyr* pyruvate, *Succ* succinate, *TAG* triacylglycerol

Cells were then cultured with [U-^13^C]glucose to determine the effects of *PDHA1* silencing on intracellular glucose metabolism. In this labeling scheme, M + 2 and M + 4 citrate arise primarily from the contribution of uniformly-labeled acetyl-CoA to the TCA cycle in one or two turns (Fig. 2a). Kinetic labeling with [U-^13^C]glucose over 4 h revealed suppression of both the M + 2 and M + 4 isotopologues (Fig. 2b), as expected. Labeling for 24 h revealed no difference in ^13^C enrichment for lactate or alanine (Additional file 1: Figure S2a, j), but significant decreases in TCA cycle intermediates and non-essential amino acids arising from TCA cycle intermediates in *PDHA1*-silenced cells (Additional file 1: Figure S2b–g). Labeling in serine and glycine was modestly increased when *PDHA1* was silenced (Additional file 1: Figure S2h, i).

Fatty-acid metabolism was examined by generating fatty acyl methyl esters from total cellular lipids and examining ^13^C distributions by GC/MS. *PDHA1* silencing substantially decreased the contribution of glucose carbon to the palmitate pool, causing a shift toward lower-order labeling and reducing labeling in the lipogenic acetyl-CoA pool by more than half (Fig. 2c). To further characterize incorporation of glucose carbon into lipids, cells were incubated with [U-^14^C]glucose, and intact lipids were separated by thin-layer chromatography (TLC). *PDHA1* silencing reduced the presence of glucose-derived carbon in total lipids and in all fractionated classes of polar and non-polar lipids (Fig. 2d).

Because shRNA against *PDHA1* expression resulted in a measurable amount of residual PDH activity (Fig. 1b), we sought to generate cells with essentially complete loss of PDH activity. To do this, we bred mice containing a *lox*P-flanked *PDHA1* exon 8 to mice expressing tamoxifen-inducible Cre recombinase (UBC-Cre-ER) and established mouse embryonic fibroblasts (MEFs) from male embryos transgenic for UBC-Cre-ER and hemizygous for the floxed *PDHA1* allele. Deletion of exon 8 is thought to impair the interaction between the E1α subunit and the rest of the complex, dramatically reducing PDH activity [39]. Tamoxifen induced deletion of *PDHA1* exon 8 in culture (Additional file 1: Figure S3a) and resulted in near-complete elimination of E1α protein and PDH activity (Additional file 1: Figure S3b, c). *PDHA1* deletion almost completely eliminated palmitate labeling from [U-^13^C]glucose (Additional file 1: Figure S3d).

### *PDHA1* silencing in H460 cells increases dependence on extracellular lipids for growth

**Fig. 3:**
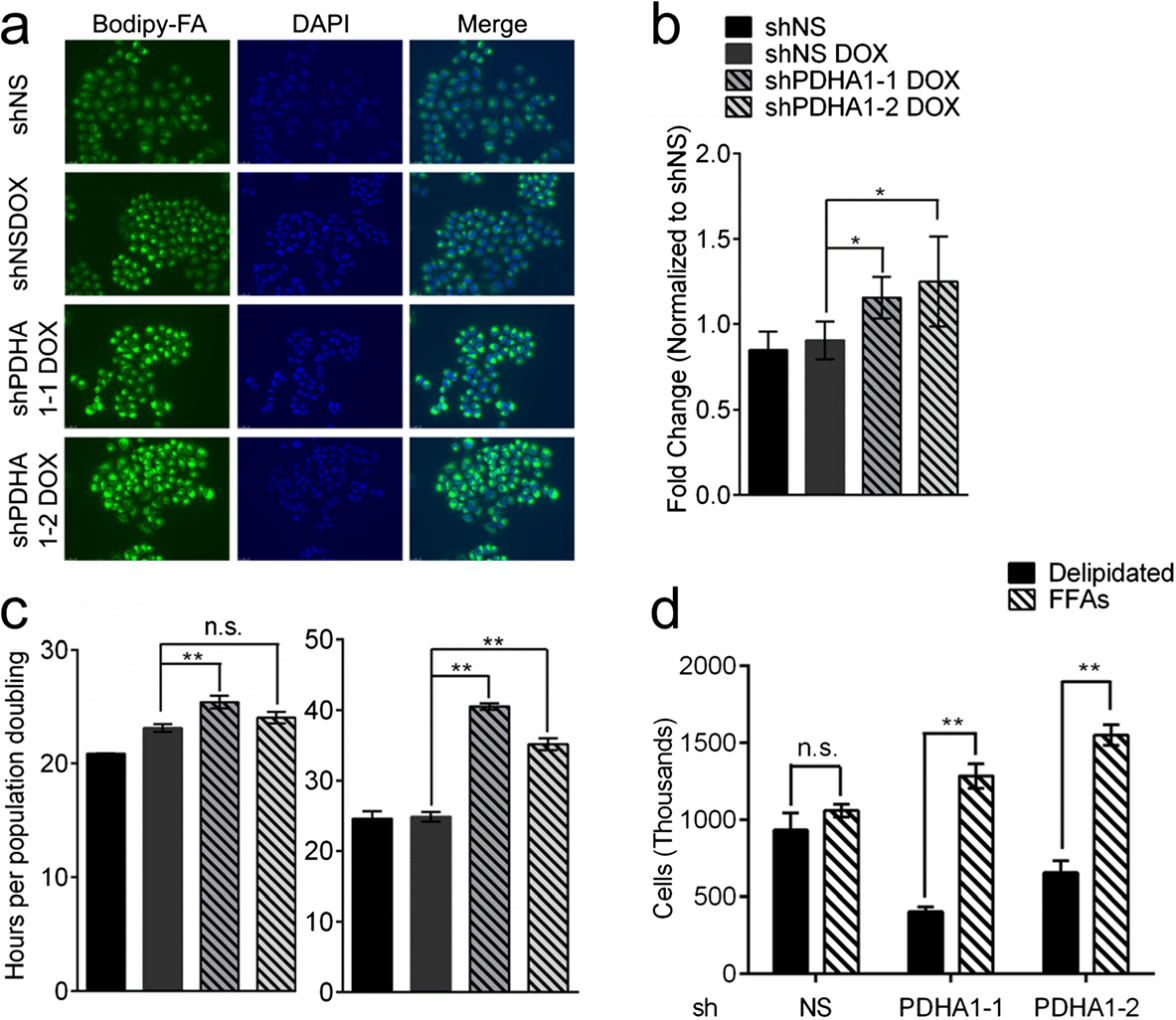
PDH suppression increases dependence on extracellular lipids for growth. **a** Uptake of BODIPY-labeled fatty acids imaged by fluorescence microscopy after 24 h of culture. **b** Quantification of BODIPY fluorescence intensity. **c** Doubling time calculated from 12-day cultures under normal (*left*) and delipidated (*right*) conditions. **d** Cell count after 4 days of culture in delipidated serum, with or without a 50 μM each of palmitate and oleate. Data are the average and SD of biological triplicates. *P < 0.05; **P < 0.005. *Abbreviations:* FFAs, free fatty acids

Despite its effects on glucose-dependent TCA cycling and lipid synthesis, *PDHA1* silencing did not significantly alter the rate of H460 cell proliferation (Additional file 1: Figure S4a). Doubling times calculated from the growth curve were, at most, only marginally increased with one of the hairpins (Fig. 3c, left). Surprisingly, even deletion of *PDHA1* exon 8 only marginally decreased the growth rate of MEFs (Additional file 1: Figure S3g), although *PDHA1*-deleted cells were smaller than their wild-type controls (Additional file 1: Figure S3f).

Oncogenic mutations in *KRAS* upregulate fatty-acid import to fuel lipid synthesis [40]. H460 cells contain an oncogenic *KRAS* mutation, *KRAS*^Q61H^ [41]. We therefore tested whether H460 cells could compensate for PDH loss by importing fatty acids from the medium. First, to maximize detection of fatty-acid uptake, cells were cultured in delipidated serum supplemented with BODIPY-labeled fatty acids. Under these lipid-deprived conditions, *PDHA1*-silenced cells demonstrated enhanced fatty-acid uptake (Fig. 3a, b). *PDHA1*-silenced cells also proliferated at a reduced rate in delipidated serum (Fig. 3c, right and Additional file 1: Figure S4b), and this growth defect was completely reversed by supplementing the medium with a mixture of palmitate and oleate (Fig. 3d). To test whether this dependence on extracellular lipids under PDH suppression could be extended to other rapidly proliferating cell lines, we used the same *PDHA1* hairpins in SFXL glioblastoma cells (Additional file 1: Figure S5a). This decreased PDH activity (Additional file 1: Figure S5b) but had no effect on cell growth in delipidated medium (Additional file 1: Figure S5c), indicating that PDH deficiency uncovers a dependence on extracellular lipids in some but not all cancer cells.

### Glutamine carbon supplies de novo lipogenesis under PDH suppression

**Fig. 4:**
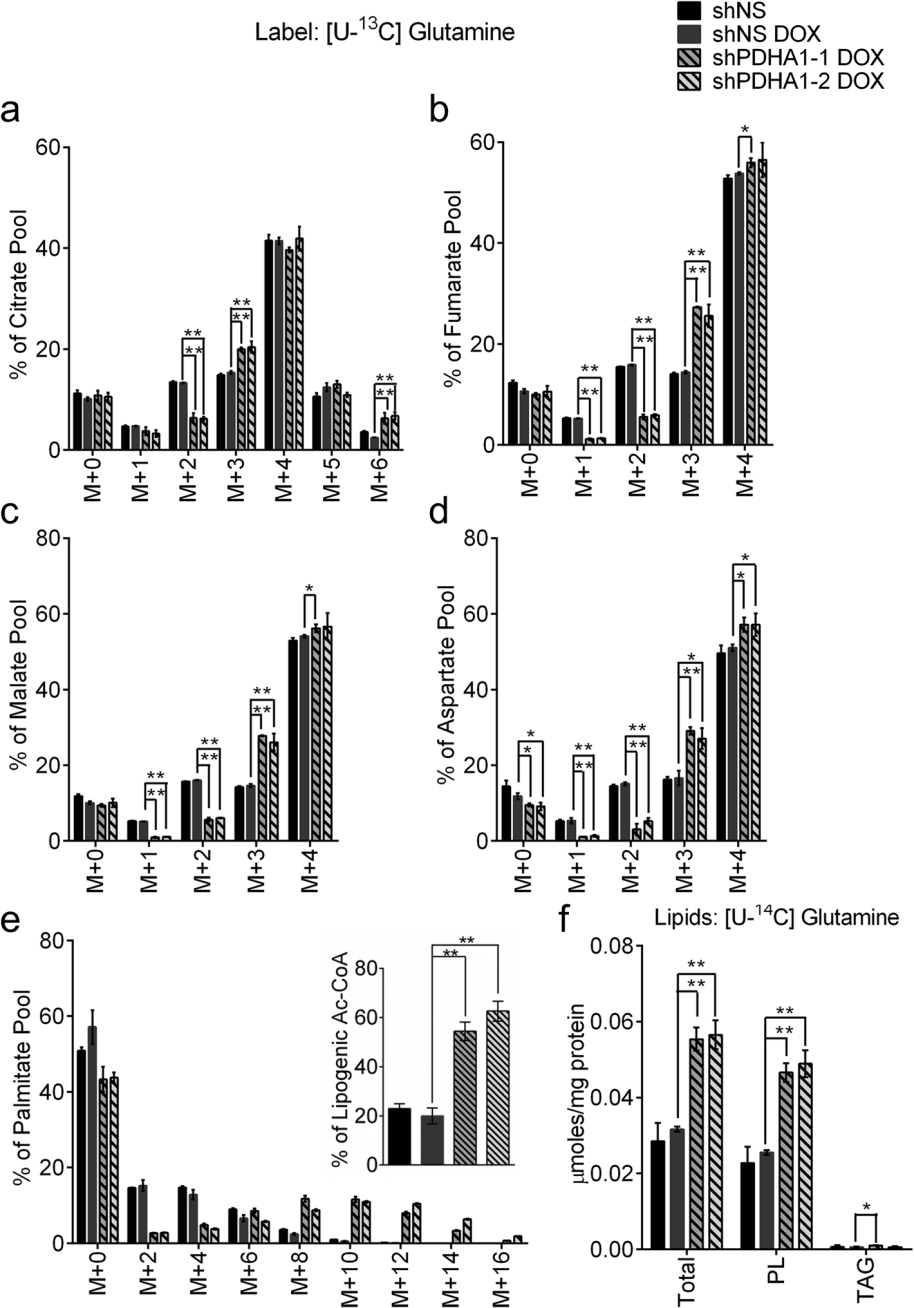
PDH suppression increases contribution of glutamine carbon to lipogenesis. Cells were cultured in medium containing glucose, [U-^13^C]glutamine, and serum for 24 h, resulting in ^13^C enrichment in **a** citrate, **b** fumarate, **c** malate, **d** aspartate, and **e** palmitate. *Inset* in **e** displays labeling in the lipogenic acetyl-CoA pool. **f** Labeling of lipid species from [U-^14^C]glutamine. Data are the average and SD of biological triplicates, except for the inset in **e**, where *error bars* represent 95 % CI. *P < 0.05; **P < 0.005. *Abbreviations*: *PL* phospholipids, *TAG* triacylglycerol

In addition to glucose, glutamine also provides a carbon source for lipogenesis through a variety of pathways [29, 42–45]. Under hypoxia, electron-transport chain dysfunction and fumarate hydratase deficiency, glutamine-derived carbon comprises the majority of lipogenic acetyl-CoA [44, 43]. We therefore determined the effects of *PDHA1* silencing on glutamine metabolism by culturing H460 cells in [U-^13^C]glutamine in lipid-replete conditions. Cells with and without the *PDHA1* shRNAs had substantial glutamine-derived labeling of TCA cycle intermediates, although the isotopologue distributions were altered when *PDHA1* was silenced (Fig. 4a–d). Furthermore, the silenced cells substantially increased the fraction of lipogenic acetyl-CoA derived from glutamine (Fig. 4e) and demonstrated enhanced contribution of glutamine-derived ^14^C to total lipids and phospholipids (Fig. 4f). *PDHA1*-deleted MEFs also demonstrated a shift from glucose to glutamine as a carbon source for palmitate synthesis (Additional file 1: Figure S3e). Together, the data indicate that PDH silencing alters metabolism of glutamine in the TCA cycle and fatty-acid synthesis. To determine the total contribution of glucose and glutamine to palmitate synthesis, H460 cells were cultured with both [U-^13^C]glucose and [U-^13^C]glutamine in a medium containing lipid-replete serum for several days. Under these conditions, when palmitate labeling reaches a steady state, the residual m + 0 fraction reports the contribution of imported lipids to the cellular palmitate pool. After 120 h of culture, the palmitate m + 0 was approximately 40 % in both control and shPDHA1-expressing cells (Additional file 1: Figure S6). This indicates that lipid uptake accounts for up to 40 % of palmitate in cellular lipids.

### A net reductive flux through cytosolic IDH provides a source of citrate for fatty-acid synthesis in *PDHA1*-silenced cells

Glutamine-dependent fatty-acid synthesis can occur through either reductive or oxidative pathways, although the latter is believed to require PDH activity [29, 43, 44]. Qualitative inspection of glutamine-derived mass isotopologues in TCA cycle intermediates demonstrated evidence of alterations in both pathways. Labeling in succinate and glutamate reflected decreased oxidative cycling of glutamine-derived carbon, evidenced by an increase in the M + 4 and M + 5 isotopologues, respectively (Additional file 1: Figure S7a, b). The small increase in citrate M + 6 is consistent with an enhanced contribution of glutamine oxidation to the acetyl-CoA pool (Fig. 4a) [29, 45]. Enhancements in citrate, fumarate, malate, and aspartate M + 3 isotopologues are consistent with altered reductive metabolism, although the expected increase in citrate M + 5 [43, 44, 46] was not observed (Fig. 4a–d).

**Fig. 5:**
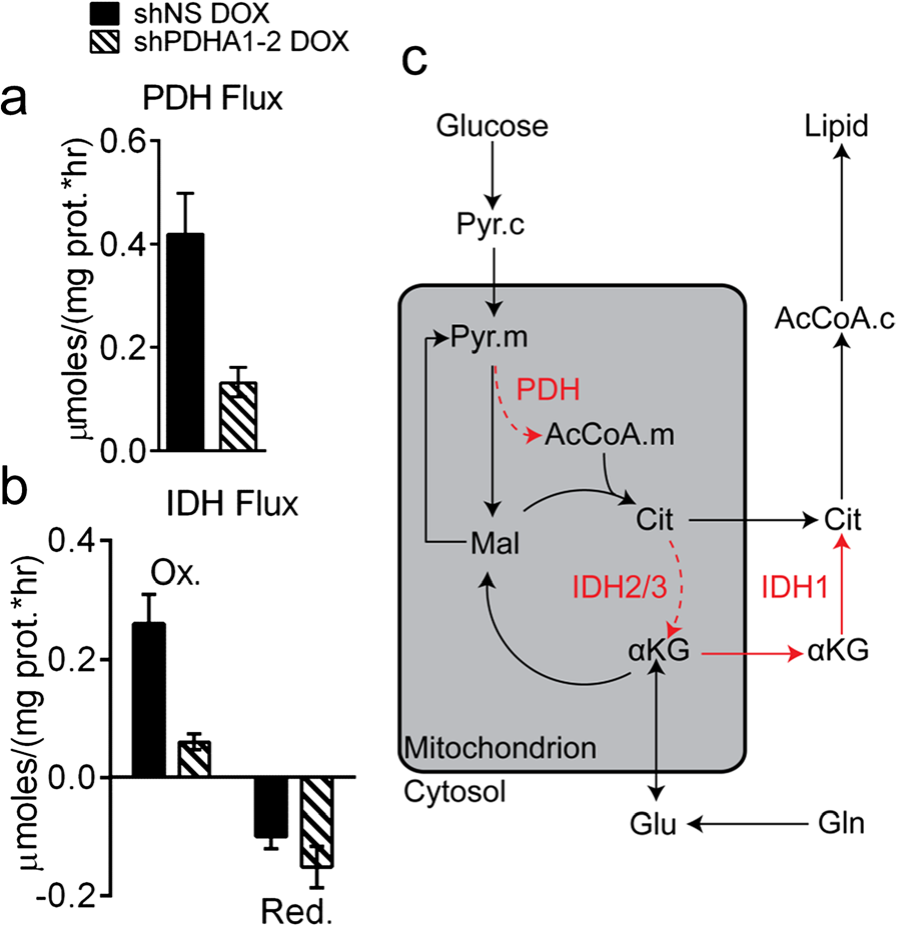
Metabolic flux analysis predicts formation of cytosolic citrate by reductive carboxylation of α-ketoglutarate. **a** Calculated PDH flux. **b** Calculated oxidative and reductive IDH fluxes. **c** Model of altered citrate metabolism in *PDHA1*-silenced cells. These changes are predicted by MFA using the INCA platform, as described in the text. Pathways in *red* are altered in *PDHA1*-silenced cells. *Solid* and *dashed* activities are predicted to be activated and suppressed, respectively. Data in panels **a** and **b** were derived from biological triplicates, with *error bars* representing 95 % CI. *Abbreviations*: *AcCoA.c* acetyl-CoA (cytosolic), *Ac.CoA.m* acetyl-CoA (mitochondrial), *αKG* α-ketoglutarate, *Cit* citrate, *Gln* glutamine, *Glu* glutamate, *Mal* malate, *Ox* oxidative, *Pyr.c* pyruvate (cytosolic), *Pyr.m* pyruvate (mitochondrial), *Red* reductive

To better understand the complex effects of *PDHA1* silencing on glutamine metabolism, experimental data in H460 cells (growth rates, metabolite uptake/secretion, ^13^C-labeled isotopologue distributions) were analyzed by Isotopomer network compartmental analysis (INCA), a form of metabolic flux analysis (MFA) that enables quantitation of many fluxes interacting with central carbon metabolism and fatty-acid synthesis [33]. The MFA approach, including reactions used in the modeling, calculated fluxes, and comparisons between simulated and experimental ^13^C distributions, is described (Additional file 1: Tables S2–S6 and Figures S9–S12). As expected, this analysis determined that *PDHA1* silencing reduced PDH flux by approximately 70 % (Fig. 5a). In control cells, ^13^C-labeling data could effectively be fit without differentiating between mitochondrial and cytosolic pools of α-ketoglutarate and citrate. The best fit indicated substantial fluxes in both the oxidative and reductive directions of IDH, with the oxidative flux exceeding the reductive flux (Fig. 5b, solid bars). However, when *PDHA1* was silenced, the model could no longer simulate the experimental data unless additional reactions were added to include compartmentalized pools of citrate and α-ketoglutarate, for example in the cytosol and mitochondria. The best fit with the new model indicated a small oxidative flux in *PDHA1*-silenced cells, significantly smaller than the oxidative IDH flux in control cells. There was also a substantial reductive IDH flux when *PDHA1* was silenced (Fig. 5b, hatched bars). According to the model, the magnitude of reductive flux exceeded the oxidative flux in *PDHA1*-silenced cells and predicted that reductive carboxylation accounted for the enhanced glutamine-derived labeling of palmitate (Fig. 5c) despite the lack of an increase in the m + 5 fraction of the total citrate pool in cells cultured with [U-^13^C]glutamine (Fig. 4a). Given the recent demonstration of the importance of pyruvate carboxylase (PC) in lung cancer cells [22], we also examined PC flux in the model. H460 cells had a measurable PC flux, but this was much smaller than the glutamine-dependent anaplerotic flux (Additional file 1: Figure S8a). *PDHA1* silencing resulted in a two- to threefold increase in PC flux (Additional file 1: Figure S8b), but this enhanced activity was still small compared to glutamine-dependent anaplerosis.

### IDH1 is required to maintain glutamine-dependent lipogenic acetyl-CoA and maximal cell proliferation under PDH suppression

**Fig. 6:**
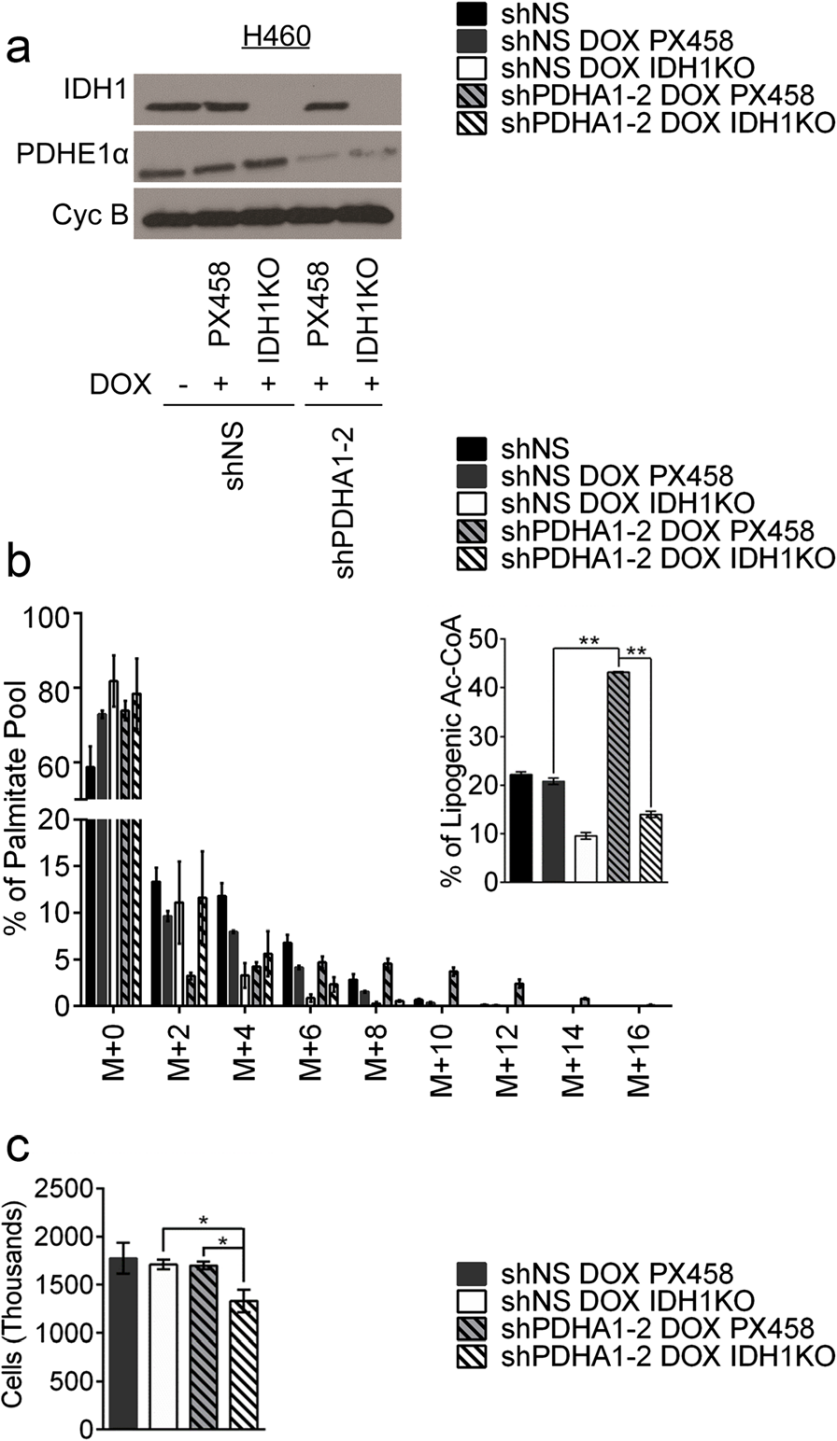
IDH1 loss suppresses contribution of glutamine carbon to lipogenesis under PDH suppression. **a** Western blot for PDH E1α and IDH1. Cyclophilin B was used as a loading control. **b** Percentage labeling in mass isotopologues of palmitate after 24 h of culture with [U-^13^C]glutamine. Inset displays labeling in lipogenic acetyl-CoA derived from glutamine carbon. **c** Cell count after 4 days of culture in normal serum. Data are the average of biological triplicates with *error bars* representing SD.

The simplest interpretation of the model in Fig. 5c is that distinct α-ketoglutarate and citrate pools arise because of subcellular compartmentalization in the mitochondria and cytosol. Considering the similarities between our system of *PDHA1* silencing and PDH suppression during hypoxia and that hypoxic cells have been reported to undergo glutamine-dependent fatty-acid synthesis through cytosolic reductive carboxylation [43], we hypothesized that enhanced reductive carboxylation occurred in the cytosol. We therefore tested whether the cytosolic isoform of IDH (IDH1) was required for the enhanced glutamine-dependent palmitate labeling during *PDHA1* silencing. CRISPR/Cas9 engineering was used to generate H460 cells containing biallelic *IDH1* mutation, then doxycycline-inducible *PDHA1* hairpins were introduced into these clones to regulate the levels of PDH E1α (Fig. 6a). Cell lines containing all combinations of IDH1 and *PDHA1* expression were cultured with [U-^13^C]glutamine. Absence of IDH1 eliminated the increased glutamine-dependent palmitate labeling during *PDHA1* silencing (Fig. 6b).

Under lipid-replete conditions, *PDHA1* silencing did not alter cell proliferation, suggesting that glutamine-derived fatty-acid synthesis might be required for maximal cell growth in the lipid-replete state. To test this hypothesis, we examined the effect of IDH1 loss on cell growth in the presence of normal or reduced PDH activity. IDH1 loss coupled with *PDHA1* silencing reduced the growth rate in lipid-replete conditions, whereas loss of either activity alone was well-tolerated (Fig. 6c). Thus, cytosolic reductive carboxylation is required for enhanced glutamine-dependent fatty-acid synthesis and maximal cell growth during suppression of PDH flux.

## Discussion

We find that activity of the PDH complex is largely dispensable for rapid cell proliferation in culture. Deletion of *PDHA1* exon 8 in mouse embryonic fibroblasts only modestly reduced the proliferative rate in complete medium, while substantial reductions in *PDHA1* expression in H460 lung cancer cells had even less impact on the doubling time unless lipids were removed from the medium. Metabolic labeling experiments revealed that when H460 cells had access to extracellular lipids, 40 % of palmitate obtained from the intracellular lipid pool contained neither glucose- nor glutamine-derived carbon. We presume that this large unlabeled palmitate pool was primarily derived from lipid uptake. Consistent with this metabolic phenotype, withdrawing lipids under PDH suppression uncovered a growth defect that was fully reversed by supplementing with physiological levels of free fatty acids. Coupled with data indicating enhanced uptake of fatty-acid tracers by *PDHA1*-silenced cells experiencing lipid depletion, this observation suggests that in some cell lines, PDH activity modestly reduces the need for extracellular lipids, but is otherwise dispensable for replicative division. Despite the high-glucose concentrations used in our experiments and the dominance of glucose as an acetyl-CoA source for lipogenesis, the cells were capable of tolerating substantial reductions in glucose-dependent lipid synthesis. It should be emphasized that *PDHA1* ablation in MEFs essentially eliminated carbon flow from glucose to fatty acids. This rules out a substantial contribution of PDH-independent accessory pathways for glucose to supply the lipogenic acetyl-CoA pool. One such mechanism described in other systems is the spontaneous decarboxylation of pyruvate in the presence of reactive oxygen species [47]. This mechanism does not produce a meaningful source of acetyl-CoA for fatty-acid synthesis in our models.

Several recent reports have described the metabolic effects of impaired mitochondrial pyruvate transport, an activity long difficult to disentangle experimentally from PDH [19, 29, 38, 45, 48, 49]. Our characterization of PDH deficiency in cultured cells identifies similarities and differences with blockade of the mitochondrial pyruvate carrier (MPC). *PDHA1* silencing, like MPC blockade, results in decreased supply of glucose to the TCA cycle and increased contribution of glutamine to fatty acids [29, 38, 45]. Both led to a small increase in glutamine consumption, and both increased aspartate abundance. However, PDH deficiency enhanced alanine levels while MPC inhibition suppressed them [29, 45]. This discrepancy is likely related to the fact that the majority of ALT activity in these models resides in the mitochondria; PDH deficiency increases the mitochondrial pyruvate pool available for transamination, whereas MPC deficiency reduces it. Finally, although both defects increased glutamine-dependent fatty-acid labeling, the mode of labeling was different. MPC deficiency resulted in enhanced labeling of mitochondrial acetyl-CoA via glutamine oxidation [29, 45]. PDH inhibition, in contrast, enhanced reductive glutamine metabolism in the cytosol.

Reductive carboxylation of α-ketoglutarate to isocitrate occurs under hypoxia or conditions of compromised function of the TCA cycle or electron-transport chain [43, 44, 46]. Isocitrate formed in this fashion can be converted to citrate and used as a carbon source for fatty-acid synthesis. In hypoxia, reductive carboxylation has been argued to result from suppression of PDH flux [43], and our data agree with this assessment because they demonstrate that PDH suppression is sufficient to induce reductive carboxylation. It has been proposed that the enhanced glutamine-dependent labeling of fatty acids in hypoxia or mitochondrial dysfunction does not represent a net reductive carboxylation flux, but rather a rebalancing of exchange fluxes across reversible IDH reactions [50]. This is an important issue, because it addresses whether reductive carboxylation accounts for true carbon flow into the fatty-acid pool. Previous efforts to model reductive fluxes considered total cellular IDH activity rather than differentiating the contributions of IDH reactions in the cytosol and mitochondria [50]. In our system of *PDHA1* silencing, adequate fits between simulations and experimental data were only achieved when distinct, compartmentalized pools of α-ketoglutarate and isocitrate/citrate were considered. The model estimated the lipogenically active cytosolic pool of citrate to be smaller than the mitochondrial pool, which accounts for the lack of an enhanced M + 5 isotopologue from glutamine in citrate under PDH suppression. Under these conditions, reductive flux exceeded oxidative flux. Experimental ablation of IDH1 in the context of *PDHA1* completely eliminated the gain in glutamine-dependent fatty-acid labeling and led to modest growth suppression. Taken together, our data indicate that IDH1 provides a net cytosolic flux of reductive carboxylation that maintains maximal cell proliferation under conditions of suppressed PDH activity.

PDH is the major gatekeeper for efficient glucose-dependent energy production and macromolecular synthesis via oxidative metabolism in the mitochondria. Yet even in MEFs, where PDH supplied some 80 % of the acetyl-CoA pool for de novo fatty-acid synthesis, near-complete elimination of PDH activity through deletion of *PDHA1* exon 8 resulted in only a 10 % increase in the doubling time. The dispensability of PDH E1α for cell proliferation emphasizes the striking metabolic flexibility in cell culture and the robustness of metabolic compensation. The ability to compensate for PDH loss is perhaps also reflected in the phenotype of male patients born with severe loss-of-function mutations in the X-linked *PDHA1* gene. These boys, some of whom exhibit loss of more than 90 % of PDH activity in primary fibroblasts, suffer from lactic acidosis and a range of neurodevelopmental abnormalities [13, 37]. However, organogenesis is largely spared, indicating that the massive amount of cell proliferation occurring during human embryonic development can also occur independently of normal PDH function.

## Conclusions

We conclude that the effects of reducing PDH activity on cell proliferation are negligible when cells have access to extracellular lipids and are able to generate lipogenic acetyl-CoA via a glutamine-dependent reductive flux through IDH1. Suppressing PDH causes cells to shift their lipogenic substrate from glucose to glutamine carbon, a process that we discovered was dependent on reductive carboxylation mediated by the cytosolic isoform of IDH (IDH1). These studies underline the plasticity of cellular metabolism with future studies being directed at ascertaining their relevance in vivo.

## Additional file

Additional file 1:
**Supplementary table and figures.** This document contains tables and supplemental figures, including details of the MFA. It consists of six tables and twelve figures.

## Abbreviations

ALT: alanine transaminase
DCA: dichloroacetate
DMEM: Dulbecco’s modified eagle medium
FBS: fetal bovine serum
IDH: isocitrate dehydrogenase
INCA: isotopomer network compartmental analysis
MEF: mouse embryonic fibroblast
MFA: metabolic flux analysis
MID: mass isotopomer distribution
MPC: mitochondrial pyruvate carrier
OAA: oxaloacetate
PDH: pyruvate dehydrogenase
PDK: pyruvate dehydrogenase kinase
TCA: tricarboxylic acid
TLC: thin-layer chromatography
