# Supplementary material for: Metabolic plasticity maintains proliferation in pyruvate dehydrogenase deficient cells
Source: Cancer Metab. 2015 Jun 29;3:7. doi: 10.1186/s40170-015-0134-4 (PMC4487196; doi:10.1186/s40170-015-0134-4)
Supplement: Additional file 1: — Supplementary table and figures. This document contains tables and supplemental figures, including details of the MFA. It consists of six tables and twelve figures. [file 40170_2015_134_MOESM1_ESM.docx]

**Supplemental Information**


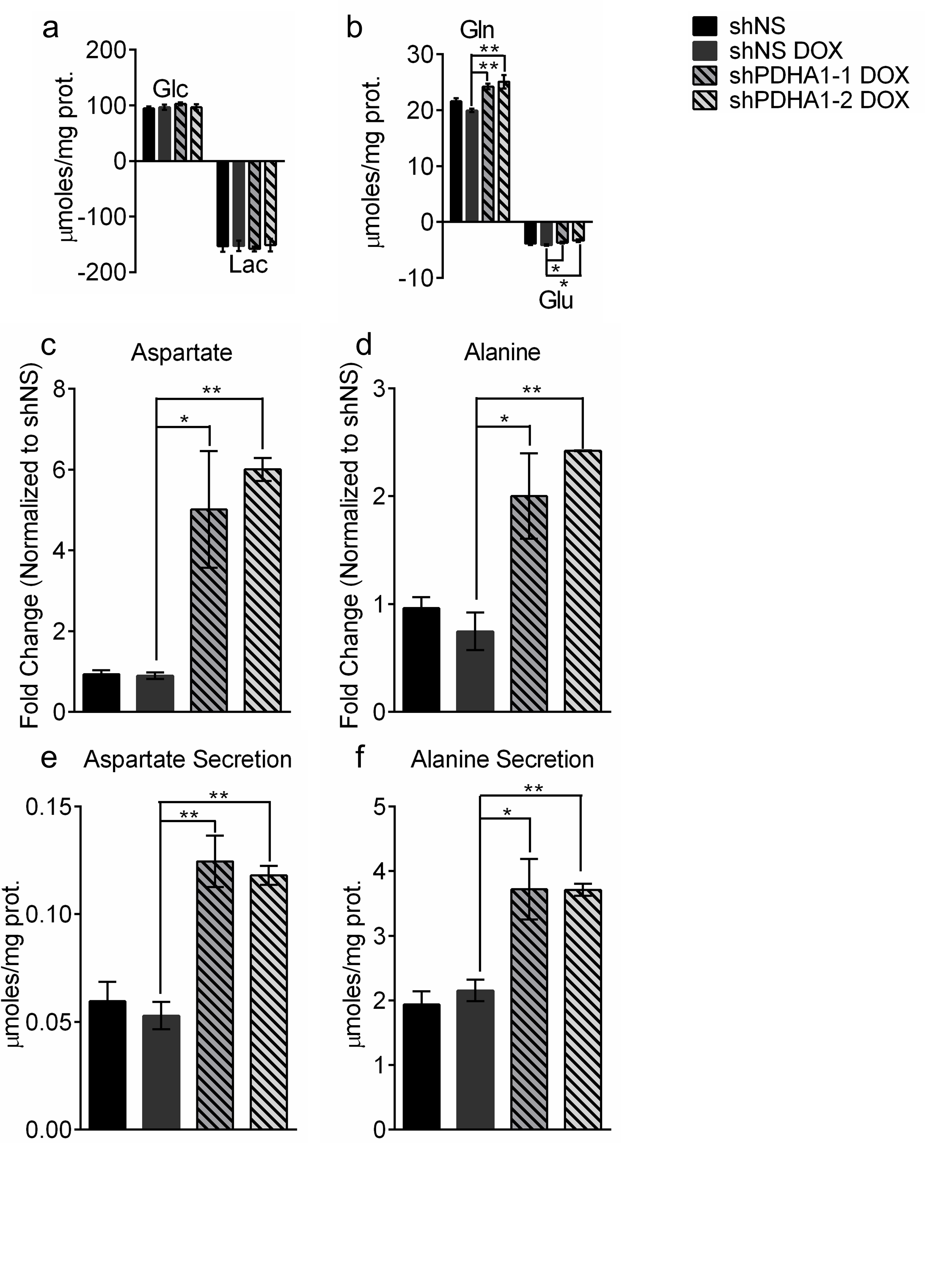


**Figure S1.** **Metabolic effects of *PDHA1* silencing in H460 cells.** (a) Glucose consumption and lactate secretion and (b) glutamine consumption and glutamate secretion in H460 cells with or without doxycycline-induced expression of shRNAs directed against *PDHA1*. Abundance of intracellular (c) aspartate and (d) alanine in cells with or without expression of *PDHA1* shRNAs. (e) Aspartate secretion and (f) alanine secretion in cells with or without expression of *PDHA1* shRNAs. Values are an average of biological triplicates with error bars representing SD. *, P<.05; **, P<.005. *Abbreviations*: Glc*,* glucose; Gln, glutamine; Glu, glutamate; Lac, lactate.


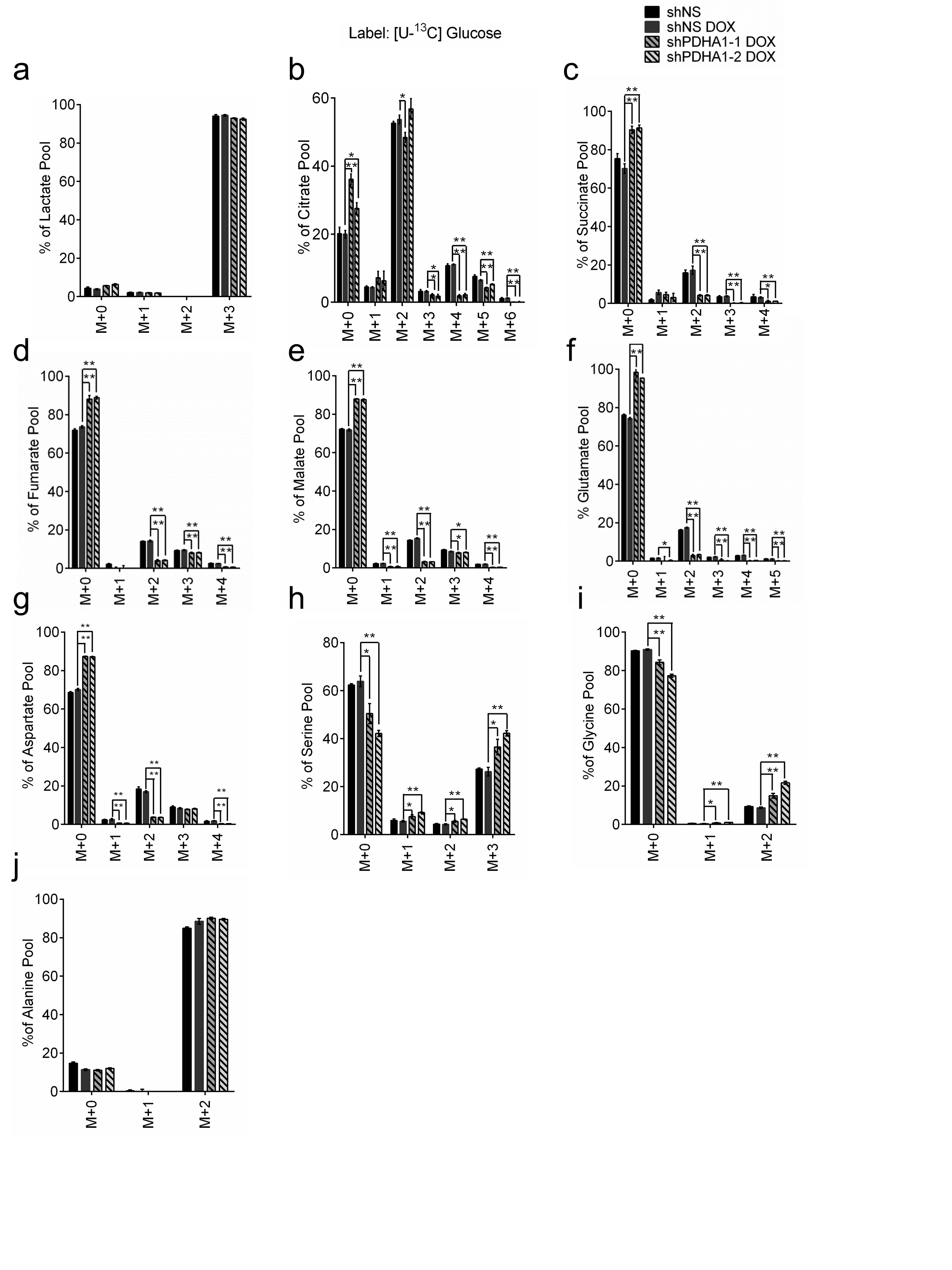


**Figure S2.** **Reduction of PDH E1α alters contribution of glucose carbon to the TCA cycle and other metabolites.** H460 cells were cultured for 24 hours in medium containing [U-^13^C]glucose, glutamine, and serum for 24 hours. Mass isotopomer distributions of (a) lactate, (b) citrate, (c) succinate, (d) fumarate, (e) malate, (f) glutamate, (g) aspartate, (h) serine, (i) glycine, and (j) alanine are shown. A two-carbon fragment of alanine was analyzed because an unrelated metabolite interfered with analysis of the entire molecule. Values are an average of biological triplicates with error bars representing SD. *, P<.05; **, P<.005.


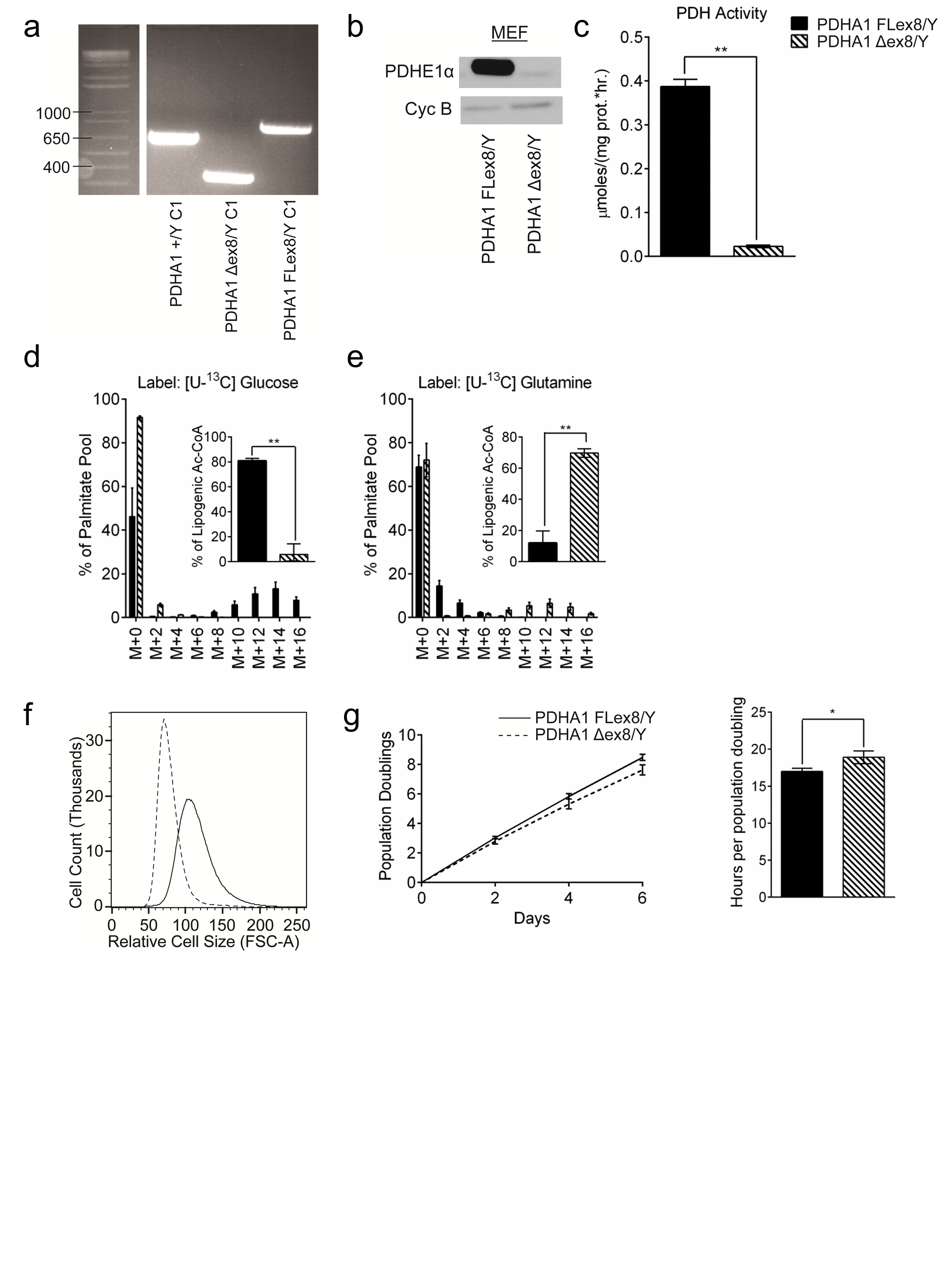


**Figure S3.** **Deletion of exon 8 of *PDHA1* in MEFs alters cell metabolism and size and marginally affects growth rate.** (a) PCR amplification of *PDHA1* gene in wild type (+/Y), exon 8 deleted (Δex8/Y), and floxed (Flex8/Y) MEFs. (b) Western blot analysis for PDH E1α. Cyclophilin B was used as a loading control. (c) Activity of pyruvate dehydrogenase was assayed *in vitro* using a dye reduction assay. Values are technical triplicates with error bars representing SD. (d) Mass isotopologues of palmitate after labeling with [U-^13^C]glucose. Inset shows calculated percentage of lipogenic acetyl-CoA derived from glucose. (e) Mass isotopologues of palmitate after labeling with [U-^13^C]glutamine. Inset shows calculated percentage of lipogenic acetyl-CoA derived from glutamine. Values are an average of biological triplicates with error bars representing SD in main panels and 95% confidence intervals in insets. (f) Relative cell size of FLex8/Y and Δex8/Y MEFs. (g) MEFs were grown in complete medium and counted every 2 days. 100,000 cells were re-plated after each passage to establish a growth curve (left) and doubling time (right). Values are an average of biological triplicates with error bars representing SD. *, P<.05; **, P<.005.


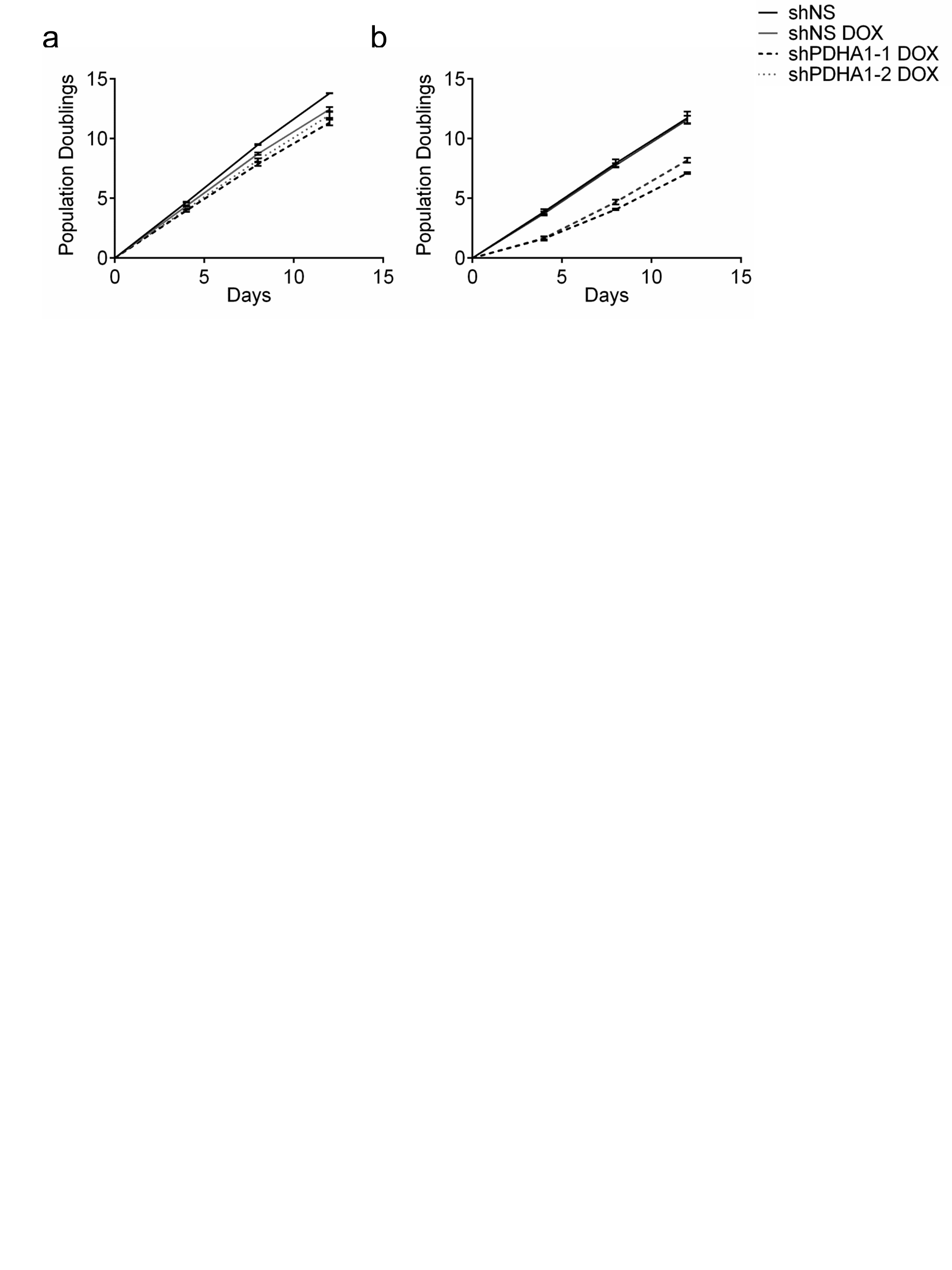


**Figure S4.** **Reduction of PDH E1α impedes growth under delipidated conditions.** H460 cells were grown in medium containing glucose, glutamine, and either normal or delipidated serum. Cells were counted every 4 days, and 100,000 cells were re-plated after each passage. Population doubling curves of cells grown in (a) normal and (b) delipidated serum. Values are an average of biological triplicates with error bars representing SD.


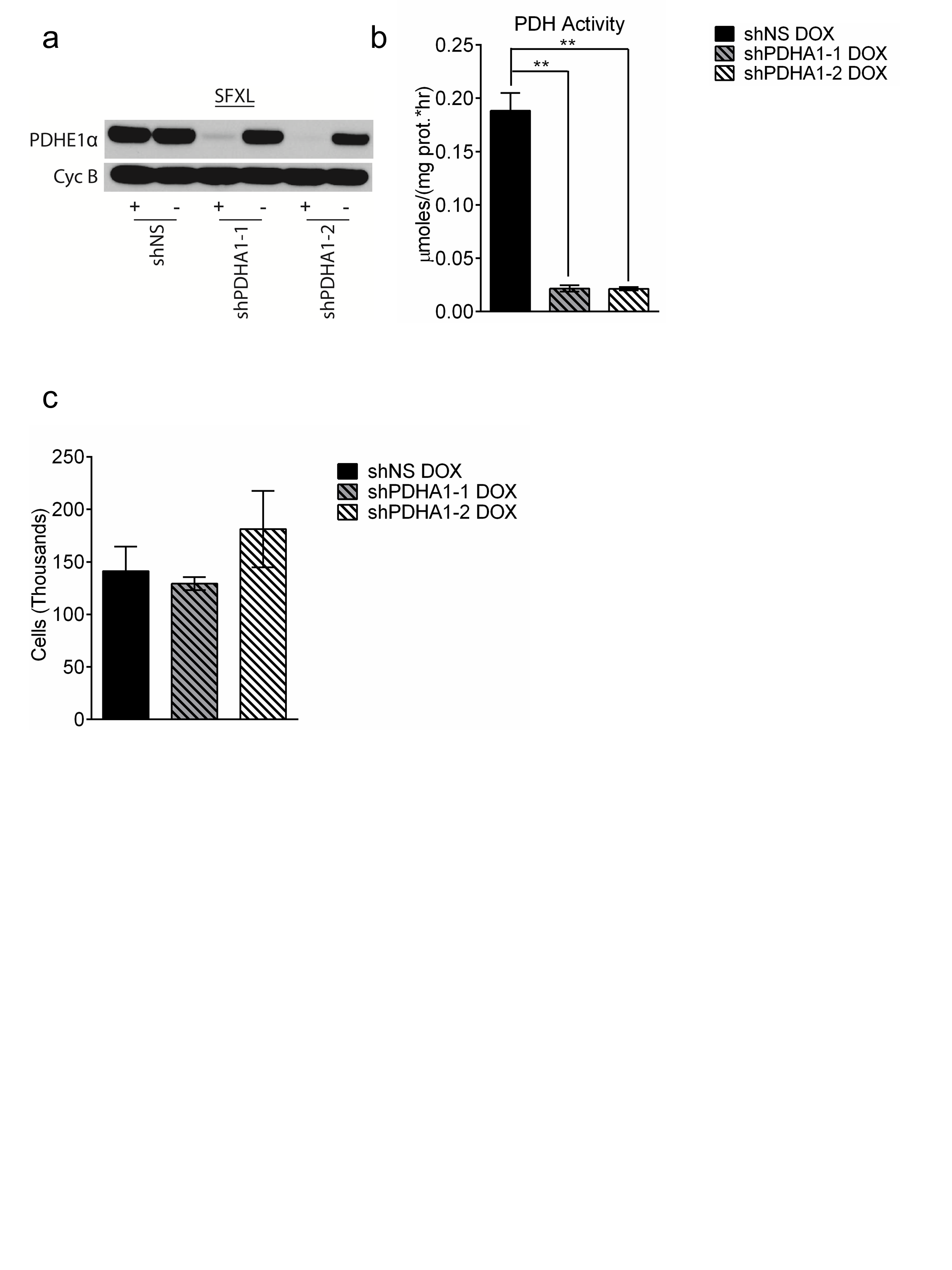


**Figure S5.** **Suppression of PDH E1α decreases PDH activity in SFXL cells.** (a) Western blot analysis for PDHE1α. Cyclophilin B was used as a loading control. (b) Activity of pyruvate dehydrogenase was assayed *in vitro* using a dye reduction assay. Values are an average of technical triplicates. (c) Cell count after 4 days of culture in delipidated conditions. Values are an average of biological triplicates. Error bars represent SD. **, P<.005.


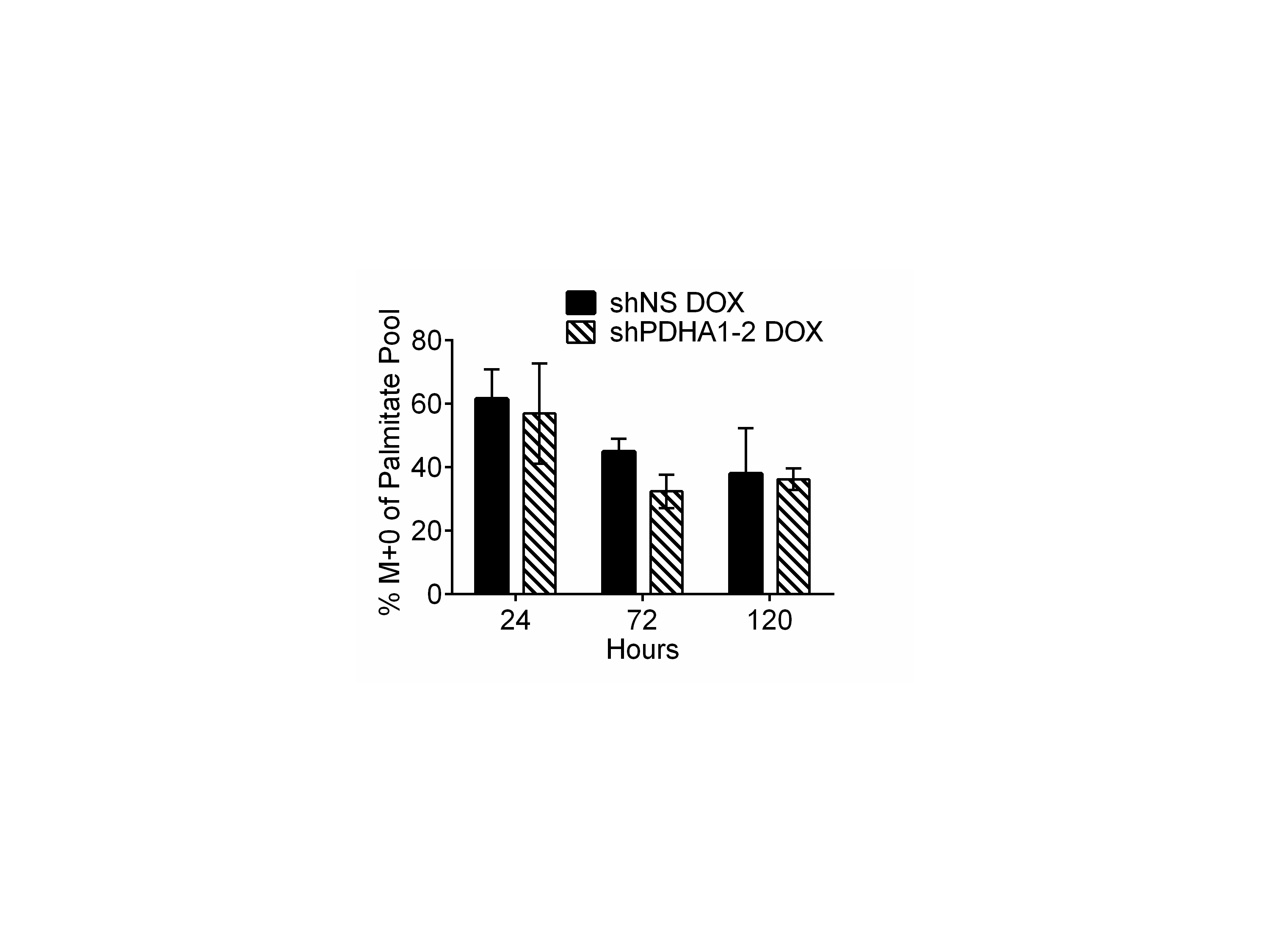


**Figure S6.** **Suppression of PDH does not alter M+0 isotopologue of palmitate when cells are incubated with [U-^13^C]glucose and [U-^13^C]glutamine.** H460 cells were grown in medium containing [U-^13^C]glucose, [U-^13^C]glutamine, and serum and total cellular lipids were extracted and analyzed at the time points above. The M+0 isotopologue of palmitate is shown.


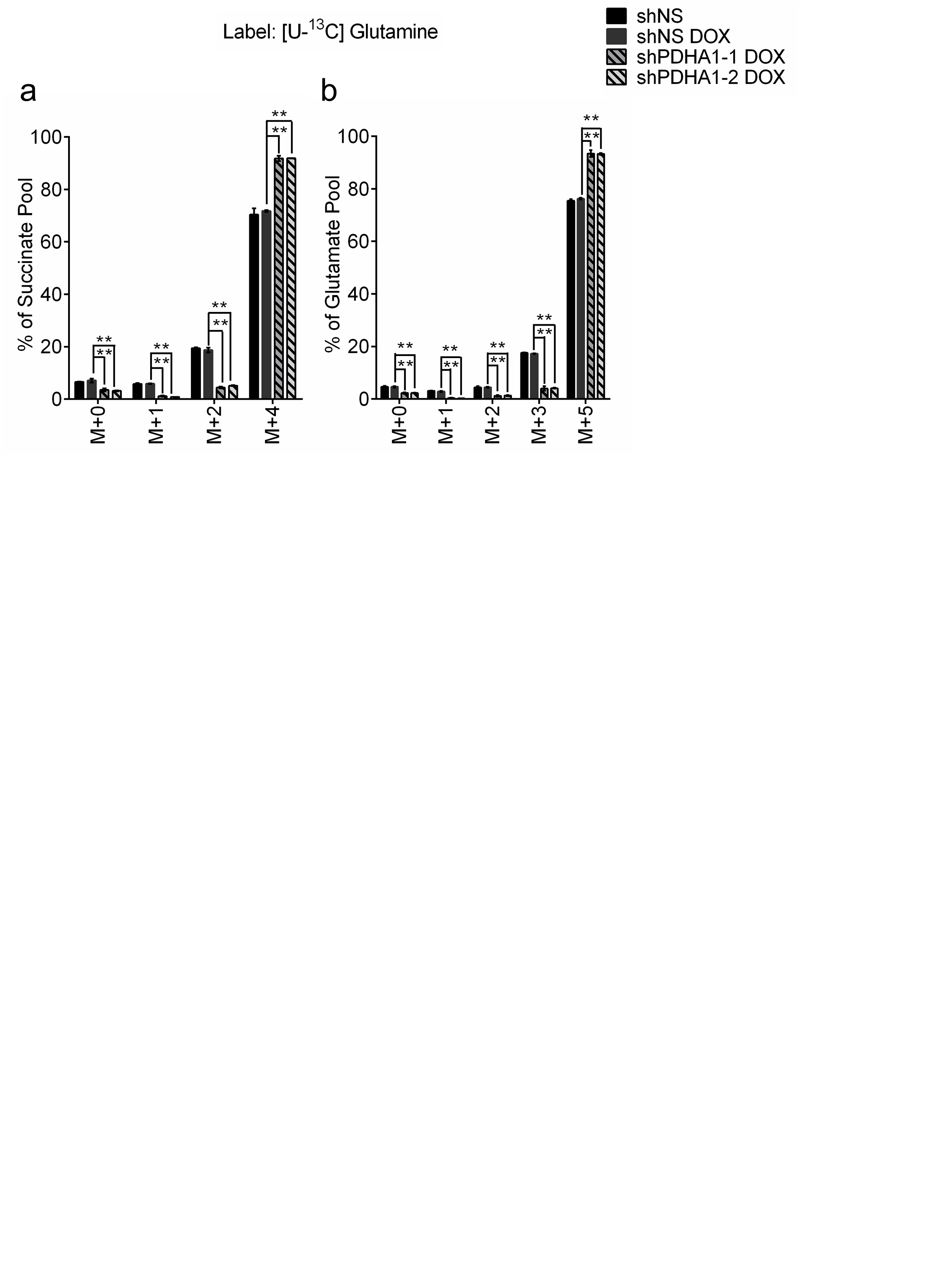


**Figure S7.** **Suppression of PDH E1α alters MIDs of succinate and glutamate when cells are incubated with [U-^13^C] glutamine.** H460 cells were incubated in medium containing glucose, [U-^13^C]glutamine, and serum for 24 hours. Mass isotopologue distributions of (a) succinate and (b) glutamate are shown. Values are an average of biological triplicates with error bars representing SD. **, P<.005.


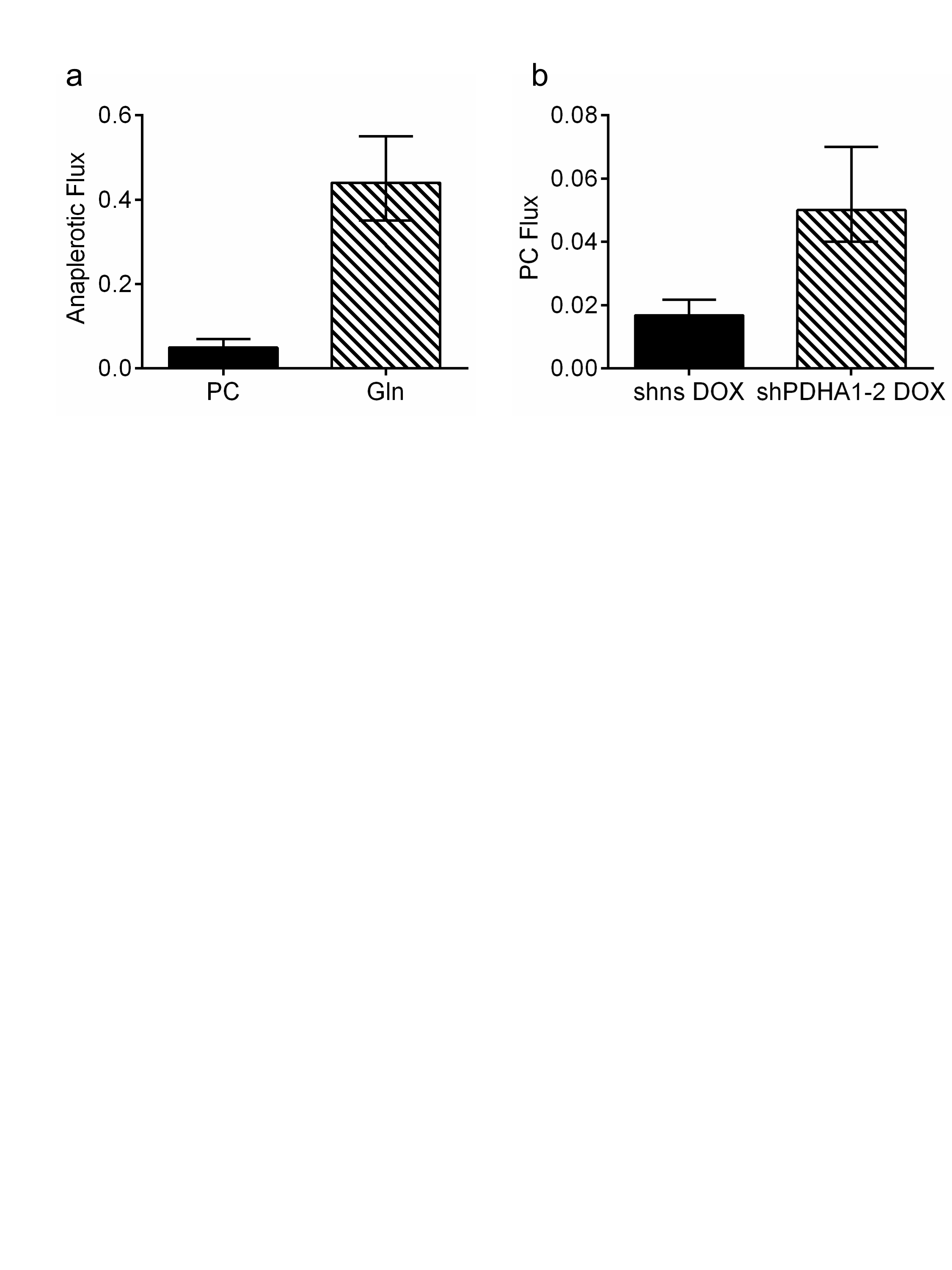


**Figure S8. Suppression of PDH induces minimal increases in PC flux.** (a) Metabolic flux analysis (MFA) estimations of PC and glutamine anaplerosis in *PDHA-1* silenced cells. (b) MFA estimations of PC flux.

**Table S1: ShRNA Sequences**

**shNS:** TGCTGTTGACAGTGAGCGCCAACAAGATGAAGAGCACCAATGTGAAGCCACA

GATGCGTTTGGTGCTCTTCATCTTGTTGTATCTACTGCCTCGGA

**shPDHA1-1:** TGCTGTTGACAGTGAGCGCTTGCTCTAGCCTGTAAGTATATAGTGAAGCCACA

GATGTATATACTTACAGGCTAGAGCAATTGCCTACTGCCTCGGA

**shPDHA1-2:** TGCTGTTGACAGTGAGCGACCGAATGGAGTTGAAAGCAGATAGTGAAGCCACA

GATGTATCTGCTTTCAACTCCATTCGGCTGCCTACTGCCTCGGA

**Metabolic Flux Analysis Procedures and Assumptions:**

Two metabolic network models describing the stoichiometry, carbon transitions, and metabolic compartmentation of H460 cells were constructed. The assumptions for these models were as follows:

1. During the experiments, cells are at metabolic steady state.
2. Cells are given 24 hours to metabolize ^13^C substrates. After 24 hours, it is assumed the isotopic labeling has reached steady state.
3. Cells grow at exponential rate.
4. ^13^CO_2_ produced during oxidation reactions is not reincorporated during carboxylation reactions.
5. The metabolites succinate and fumarate are symmetrical and their metabolism through the TCA cycle does not produce a particular orientation.
6. The biomass requirements for CHO cells and B-cells, which have been extensively characterized [[1](#_ENREF_1), [2](#_ENREF_2)], are similar to the cells used here.
7. The metabolites pyruvate, acetyl-CoA, malate, aspartate, and oxaloacetate are metabolically active in both the cytosol and mitochondria. Malate, aspartate, and oxaloacetate are allowed to freely mix between the compartments.
8. For flux calculations in cells expressing *PDHA1* shRNAs, it was not possible to reconcile the palmitate and citrate labeling from [U-^13^C_5_]glutamine by modeling a single pool of citrate produced from citrate synthase and the reductive carboxylation of α-ketoglutarate. To account for this discrepancy, two separate pools of citrate were modeled in *PDHA1*-silenced cells. One citrate pool (Cit.m) is produced in the mitochondria via citrate synthase and can be oxidized through isocitrate dehydrogenase. A second, independent citrate pool (Cit.c) is produced through reductive carboxylation. Both pools contribute to lipogenic acetyl-CoA production. This cytosolic citrate is the same citrate pool as the cytosolic citrate formed from mitochondrial citrate export (Figure 5c). However, the citrate produced from reductive carboxylation does not have to mix with the mitochondrial citrate pool directly in our model. This one cytosolic citrate pool is then used to make lipogenic 2 carbon units.
9. During the extraction process, intracellular pools of metabolites are homogenized. Therefore GC-MS analysis of the isotopic enrichment of these metabolites reflects the mixture of distinct metabolic pools. By employing the INCA platform to perform metabolic flux analysis, it is possible to extract meaningful information from these mixed pools [[3](#_ENREF_3)]. To do this, the model employs parameters to account for the mixing of mitochondrial and cytosolic metabolites.

The primary way the model accounts for the mixing of distinct pools is through the inclusion of ‘pseudo’ fluxes. These parameters allow for the mixing of material without impacting the true net flow of material through the metabolic network. To properly utilize these parameters, we must define a metabolite pool that represents the sampled pool due to the mixing of separate compartments during the extraction process. For example, the model defines a sampled aspartate pool as Asp. It represents the mixing of both mitochondrial (Asp.m) and cytosolic (Asp.c) pools. These pools can mix in the model (as a true flux) as Asp.m 🡨🡪 Asp.c, which allows aspartate to exchange between the mitochondria to the cytosol. During extraction, both Asp.m and Asp.c combine to form Asp (our sampled fraction). The use of zero coefficients (0*Asp.m🡪Asp) allow one to model compartmentalization without impacting the net fluxes of the system [[4](#_ENREF_4)]. The ‘flux’ of this making is then set to 100 (Cit🡪Sink, Asp🡪Sink=100) to allow for a convenient estimation of the contribution of each pool to the metabolic network. Using these parameters it is not necessary to know the fractional contribution of each compartment prior to flux modeling.

For all fluxes, the sum-of-squared residuals (SSR) of the best fit model, the degrees of freedom (DOF), and the lower and upper bounds of 95% confidence intervals are reported. For reversible fluxes, the exchange flux is presented as $v_{exch}^{[0,100]}=100\times\frac{v_{exch}}{v_{exch}+v_{ref}}$, where $v_{ref}$ is the citrate synthase flux value [[4](#_ENREF_4)].

Table S2. Measured GC-MS ions used for metabolic flux analysis.

| **Metabolite** | **Mass** | **Composition** | **Carbons** | **Standard Error of the Mean (mol%)** | | | |
| --- | --- | --- | --- | --- | --- | --- | --- |
|  |  |  |  | **shNS**  **DOX ^13^Glc** | **shNS**  **DOX ^13^Gln** | **shPDHA**  **1-2 DOX ^13^Glc** | **shPDHA**  **1-2 DOX ^13^Gln** |
| Lac | 219 | C_8_H_19_O_3_Si_2_ | 1 2 3 | 0.9 | 0.9 | 0.9 | 0.9 |
| Cit | 465 | C_17_H_37_O_7_Si_4_ | 1 2 3 4 5 6 | 0.8 | 0.8 | 1.3 | 0.8 |
| Fum | 245 | C_9_H_17_O_4_Si_2_ | 1 2 3 4 | 0.9 | 0.9 | 0.9 | 1.8 |
| Mal | 335 | C_12_H_27_O_5_Si_3_ | 1 2 3 4 | 0.5 | 0.5 | 0.5 | 1.8 |
| Mal | 233 | C_9_H_21_O_3_Si_2_ | 2 3 4 | 0.5 | 0.5 | 0.5 | 1.3 |
| Asp | 334 | C_12_H_28_NO_4_Si_3_ | 1 2 3 4 | 0.5 | 0.9 | 0.5 | 1.4 |
| Glu | 363 | C_14_H_33_NO_4_Si_3_ | 1 2 3 4 5 | 0.5 | n/a | 0.5 | 0.6 |
| PA | 270 | C_17_H_34_O_2_ | 1-16 | 1.6 | 2.7 | 1.3 | 3.0 |

**Table S3. Reactions used for flux modeling of shNS DOX H460 cells.** *No citrate compartmentalization*.

| **Pathway** | | | | |  | | **Reaction Name** |
| --- | --- | --- | --- | --- | --- | --- | --- |
| **Glycolysis** | |  | |  | | |  |
| Glucose (abcdef) | | -> | | G6P (abcdef) | | | HK |
| G6P (abcdef) | | -> | | F6P (abcdef) | | | PGI |
| F6P (abcdef) | | -> | | DHAP (cba) + GAP (def) | | | PFK |
| DHAP (abc) | | <-> | | GAP (abc) | | | TPI |
| GAP (abc) | | <-> | | 3PG (abc) | | | GAPDH |
| 3PG (abc) | | <-> | | PEP (abc) | | | ENO |
| PEP (abc) | | -> | | Pyr.c (abc) | | | PK |
| Pyr.c (abc) | | -> | | Lac (abc) | | | LDH |
| Pyr.c (abc) | | -> | | Pyr.m (abc) | | | Pyr Transport |
| **TCA Cycle** | |  | |  | | |  |
| Pyr.m (abc) | | -> | | AcCoA.m (bc) + CO_2_ (a) | | | PDH |
| AcCoA.m (ab) + OAA.m (cdef) | | -> | | Cit (fedbac) | | | CS |
| Cit (abcdef) | | <-> | | aKG (abcde) + CO_2_ (f) | | | IDH |
| aKG (abcde) | | -> | | Suc ( ½ bcde + ½ edcb) + CO_2_ (a) | | | ADH |
| Suc ( ½ abcd + ½ dcba) | | <-> | | Fum ( ½ abcd + ½ dcba) | | | SDH |
| Fum ( ½ abcd + ½ dcba) | | <-> | | Mal.m (abcd) | | | FUM |
| Mal.m (abcd) | | <-> | | OAA.m (abcd) | | | MDH.m |
| **Anaplerosis/Cataplerosis** | | | | | | |  |
| Gln (abcde) | | -> | | Glu (abcde) | | | GLS |
| Glu (abcde) | | <-> | | aKG (abcde) | | | GDH |
| Pyr.m (abc) + CO_2_ (d) | | -> | | OAA.m (abcd) | | | PC |
| Mal.m (abcd) | | -> | | Pyr.m (abc) + CO_2_ (d) | | | ME.m |
| Mal.c (abcd) | | -> | | Pyr.c (abc) + CO_2_ (d) | | | ME.c |
| 3PG (abc) | | -> | | Ser (abc) | | | PP |
| OAA.m (abcd) | | <-> | | Asp.m (abcd) | | | GOT2 |
| Asp.m (abcd) | | <-> | | Asp.c (abcd) | | | Asp Transport |
| Glu (abcde) | | -> | | Pro (abcde) | | | OC |
| Cit (abcdef) | | -> | | AcCoA.c (ed) + OAA.c (fcba) | | | ACL |
| OAA.c (abcd) | | <-> | | Mal.c (abcd) | | | MDH.c |
| Pyr.m (abc) | | -> | | Ala (abc) | | | GPT |
| **Biomass** | |  | |  | | |  |
| 0.9*Asp.c (abc) + 0.97*Glu.c (defgh) + 1.51*Ala (ijk) + 0.81*Gln (lmnop) + 6.14*AcCoA.c (qr) + 1.08*Ser (stu) + 0.788*Pro (vwxyz) + 0.29*DHAP (123)🡪Biomass | | -> | | Biomass | | | Growth |
| **PA synthesis** | |  | |  | | |  |
| 0*AcCoA.c (ab) + 0*AcCoA.c (cd) + 0*AcCoA.c (ef) + 0*AcCoA.c (gh) + 0*AcCoA.c (ij) + 0*AcCoA.c (kl) + 0*AcCoA.c (mn) + 0*AcCoA.c (op) | | -> | | Palm (abcdefghijklmnop) | | | FAS |
| 0*AcCoA.x (ab) + 0*AcCoA.x (cd) + 0*AcCoA.x (ef) + 0*AcCoA.x (gh) + 0*AcCoA.x (ij) + 0*AcCoA.x (kl) + 0*AcCoA.x (mn) + 0*AcCoA.x (op) | | -> | | Palm (abcdefghijklmnop) | | | FAS |
| Palm.d (abcdefghijklmnop) | | -> | | Palm (abcdefghijklmnop) | | |  |
|  | |  | |  | | |  |
| **Compartmentalization/Mixing** | |  | |  | | |  |
| 0*Mal.m (abcd) | | -> | | Mal (abcd) | | |  |
| 0*Mal.c (abcd) | | -> | | Mal (abcd) | | |  |
| Mal (abcd) | | -> | | Sink | | | Fixed to 100 |
| Mal.m (abcd) | | <-> | | Mal.c (abcd) | | |  |
| 0*Asp.m (abcd) | | -> | | Asp (abcd) | | |  |
| 0*Asp.c (abcd) | | -> | | Asp (abcd) | | |  |
| Asp (abcd) | | -> | | Sink | | | Fixed to 100 |
| Asp.m (abcd) | | <-> | | Asp.c (abcd) | | |  |
|  | |  | |  | | |  |
| **Extracellular Fluxes** | | |  | | |  | |
| Glucose.x (abcdef) | -> | | Glucose (abcdef) | | | Glucose Uptake | |
| Lac (abc) | -> | | Lac.x (abc) | | | Lactate Secretion | |
| Gln.x (abcde) | -> | | Gln (abcde) | | | Gln Uptake | |
| Glu.c (abcde) | <-> | | Glu.x (abcde) | | | Glu Secretion | |
| Ala (abc) | -> | | Ala.x (abc) | | | Ala Secretion | |
| Asp.c (abc) | -> | | Asp.x (abc) | | | Asp Secretion | |

**Table S4. Additional reactions for flux modeling of shPDHA1-2 DOX H460 cells.** In order to match the labeling in palmitate from ^13^C glutamine, it was necessary to model two separate pools of citrate. This was accomplished by adding the following reactions to the network model.

| **Citrate Modeling** |  |  |  |
| --- | --- | --- | --- |
| Cit.m (abcdef) | -> | Cit.c (abcdef) | Cit Transport |
| Cit.m (abcdef) | -> | aKG.m (abcde) + CO_2_ (f) | Oxidative IDH |
| aKG.c (abcde) | <-> | aKG.m (abcde) | aKG Transport |
| aKG.c (abcde) + CO_2_ (f) | -> | Cit.c (abcdef) | Reductive IDH |
| Cit.c (abcdef) | -> | AcCoA.c (ed) + OAA.c (fcba) | ACL |
| 0*Cit.d (abcdef) | -> | Cit.m (abcdef) | Citrate Dilution |
| 0*Cit.c (abcdef) | -> | Cit (abcdef) | Citrate Mixing |
| 0*Cit.m (abcdef) | -> | Cit (abcdef) | Citrate Mixing |
| Cit (abcdef) | -> | Sink | Fix to 100 |

*Abbreviations*:

Enzymes**:** HK, hexokinase; PI, phosphoglucose isomerase; PFK, phosphofructokinase; TPI, triose phosphate isomerase; GAPDH, glyceraldehyde 3 phosphate dehydrogenase; ENO, enolase; PK, pyruvate kinase; LDH, lactate dehydrogenase; PDH, pyruvate dehydrogenase; CS; citrate synthase; IDH, isocitrate dehydrogenase; ADH, a-ketoglutarate dehydrogenase; SDH, succinate dehydrogenase; FUM, fumarase; MDH, malate dehydrogenase; ME, malic enzyme; PC, pyruvate carboxylase; ACL, ATP citrate lyase; GDH, glutamate dehydrogenase, GOT, glutamate oxaloacetate transaminase; GLS, glutaminase; GPT, glutamate pyruvate transaminase; PP, phosphoserine phosphatase; OC, ornithine cyclodeaminase; FAS, fatty acid synthase.

Metabolites**:** AcCoA, acetyl-CoA; aKG, a-ketoglutarate dehydrogenase; Ala, alanine; Asp, aspartate; Cit, citrate; DHAP, dihydroxyacetone phosphate; Fum, fumarate; F6P, fructose-6-phosphate; GAP, glyceraldehyde-3-phosphate; 3PG, 3-phosphoglycerate; Glu, glutamate; Gln, glutamine; G6P, glucose-6-phosphate; Lac, lactate; Mal, malate; OAA, oxaloacetate; Palm, palmitate; PEP, phosphoenolpyruvate; Pro, proline; Pyr, pyruvate; Ser, serine.

**Table S5. Calculated fluxes for shNS DOX H460 cells during exponential growth.** All units are μmol/hr/mg protein. Exchange fluxes are scaled 0-100% with reference to citrate synthase. SSE=69.4 with expected SSE [48.8, 95], DOF= 70.

| **Net Flux** | **Value** | **95% Confidence interval** | |
| --- | --- | --- | --- |
| **Glycolysis** |  |  |  |
| Glucose Uptake | 4.85 | [4.41, | 5.28] |
| HK | 4.85 | [4.41, | 5.28] |
| PGI | 4.85 | [4.41, | 5.28] |
| PFK | 4.85 | [4.41, | 5.28] |
| TPI | 4.85 | [4.41, | 5.28] |
| GAPDH | 9.68 | [8.81, | 10.55] |
| ENO | 9.64 | [8.77, | 10.51] |
| PK | 9.64 | [8.77, | 10.51] |
| LDH | 9.08 | [8.21, | 9.95] |
| Lac Secretion | 9.08 | [8.21, | 9.95] |
| Pyr Transport | 0.56 | [0.48, | 0.63] |
| **TCA Cycle** |  |  |  |
| PDH | 0.42 | [0.35, | 0.50] |
| CS | 0.42 | [0.34, | 0.50] |
| IDH | 0.16 | [0.13, | 0.19] |
| ADH | 0.25 | [0.21, | 0.29] |
| SDH | 0.25 | [0.21, | 0.29] |
| FUM | 0.25 | [0.21, | 0.29] |
| MDH.m | 0.45 | [0.37 | 0.53] |
| **Anaplerosis/Cataplerosis** | |  |  |
| Gln Uptake | 0.56 | [0.47, | 0.65] |
| Gls | 0.53 | [0.44, | 0.61] |
| GDH | 0.09 | [0.06, | 0.12] |
| ME.m | 0.06 | [0.04, | 0.09] |
| ME.c | 0.0 | [0.0, | 0.03] |
| MDH.C | 0.26 | [0.21, | 0.32] |
| PC | 0.02 | [0.01, | 0.02] |
| PP | 0.05 | [0.04, | 0.06] |
| ACL | 0.26 | [0.21, | 0.32] |
| GOT2 | 0.04 | [0.04, | 0.05] |
| GPT | 0.18 | [0.16, | 0.19] |
| OC | 0.03 | [0.03, | 0.04] |
| Biomass | 0.04 | [0.03, | 0.05] |

| **Exchange Flux** |  |  |  |
| --- | --- | --- | --- |
| IDH | 20.02 | [17.44, | 23.12] |
| LDH | 97.96 | [0, | 100] |
| TPI | 3.14 | [0, | 100] |
| GAPDH | 98.9 | [0, | 100] |
| ENO | 98.9 | [0, | 100] |
| SDH | 18.7 | [0.00, | 100] |
| FUM | 100.00 | [88.2, | 100] |
| MDH.m | 62.30 | [35.65, | 89.5] |
| GDH | 90.22 | [85.31, | 94.69] |
| MDH.C | 90.05 | [0, | 100] |
| GOT2 | 75.37 | [0, | 100] |
| **Additional Extracellular Fluxes** | |  |  |
| Glu Secretion | 0.4 | [0.33, | 0.49] |
| Ala Secretion | 0.11 | [0.10, | 0.12] |
| Asp Secretion | 0.0027 | [0.0025, | 0.0029] |
| **Compartmentalization/Mixing** | |  |  |
| Mal.m -> Mal | 100 | [0, | 100] |
| Mal.c -> Mal | 0 | [0, | 100] |
| Mal -> Sink | 100 | [100, | 100] |
| Mal.m <-> Mal.c (Net) | -0.26 | [-0.3, | -0.2] |
| Mal.m <-> Mal.c (Exchange) | 90.5 | [0, | 100] |
| Asp.m -> Asp | 99.57 | [0, | 100] |
| Asp.c -> Asp | 0.43 | [0, | 100] |
| Asp -> Sink | 100 | [100, | 100] |
| Asp.m <-> Asp.c (Net) | 0.04 | [0.03, | 0.05] |
| Asp.m <-> Asp.c (Exchange) | 78.5 | [0, | 100] |

**
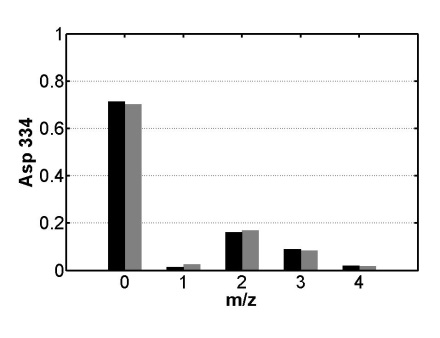

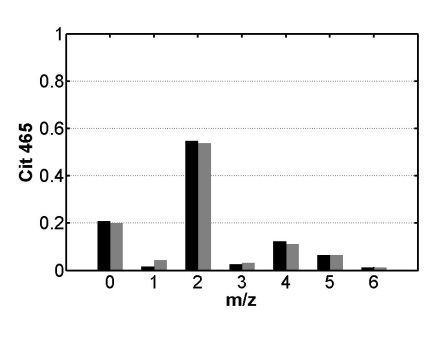

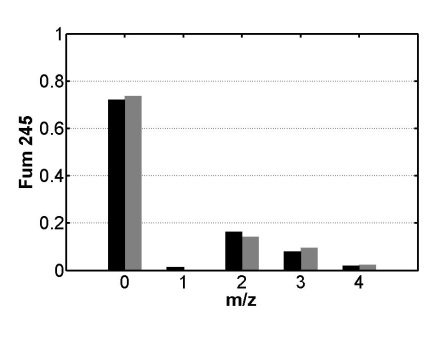

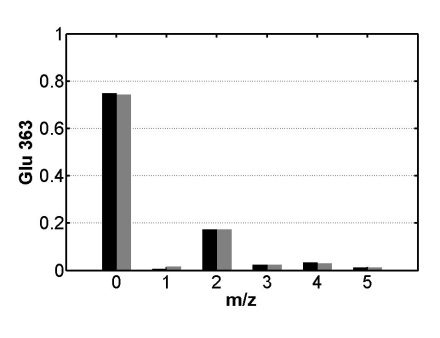

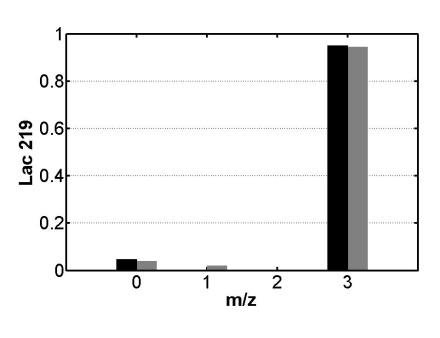

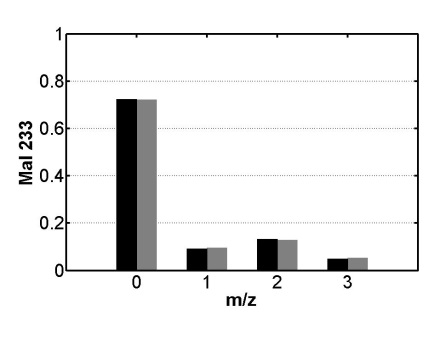

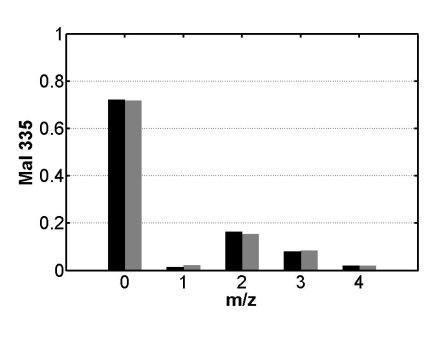

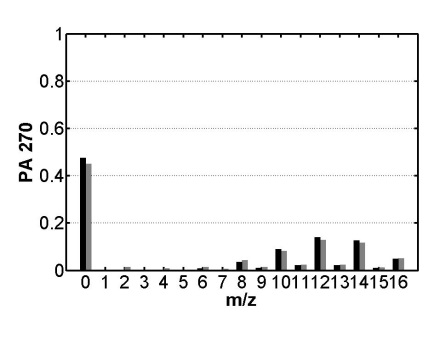

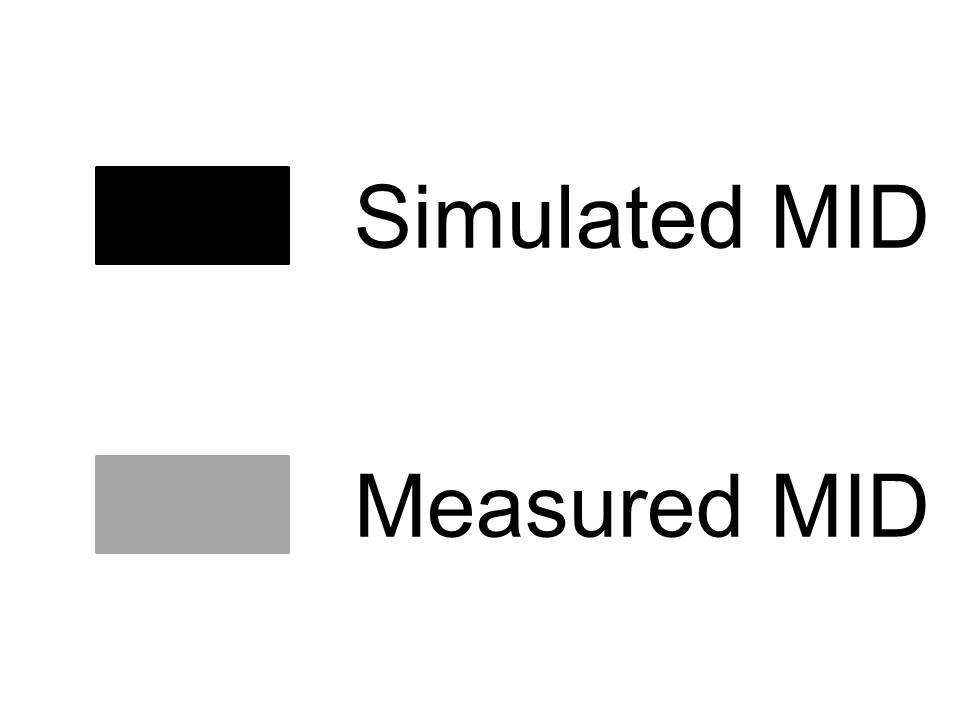
**

**Figure S9. Simulated and measured MIDs for shNS DOX H460 cells fed ^13^C glucose.** The data shown has been corrected for natural abundance. The MIDs were combined with ^13^C glutamine data to simulate fluxes in Table S5.

**
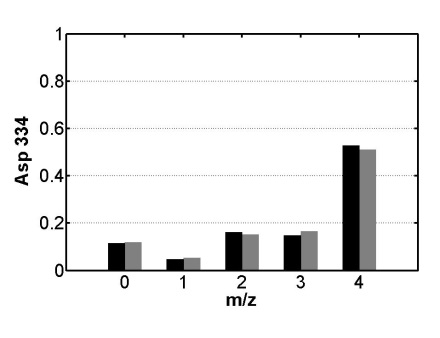

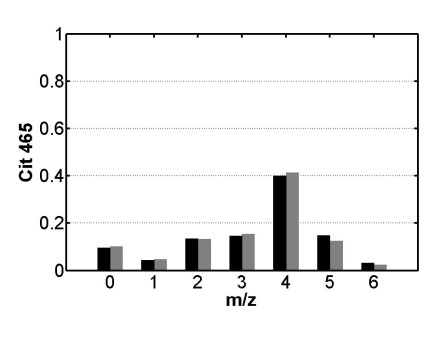

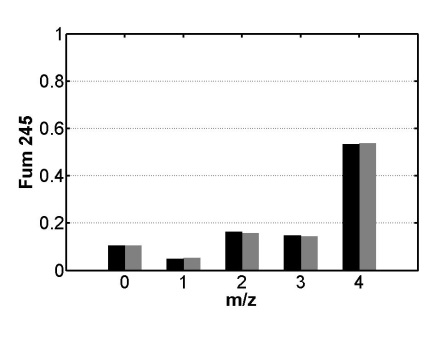

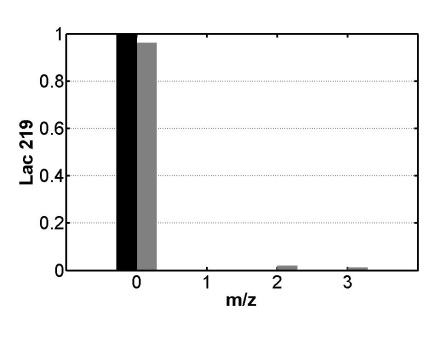

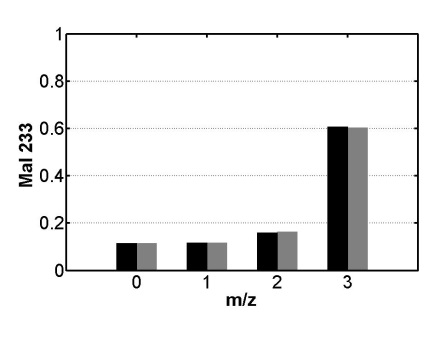

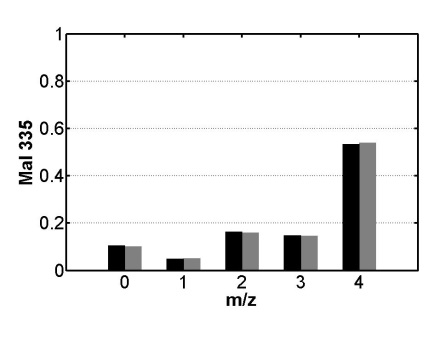

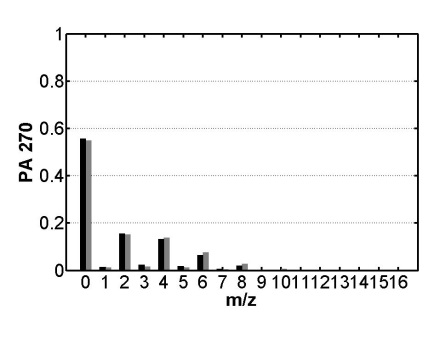

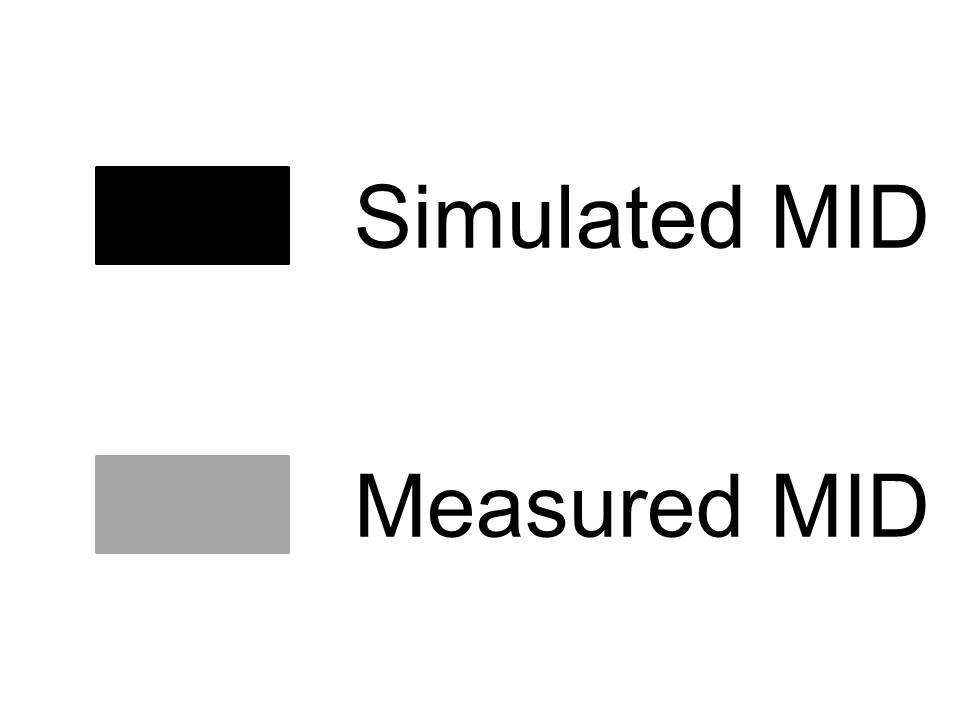
**

**Figure S10. Simulated and measured MIDs for shNS DOX H460 cells fed ^13^C glutamine.** The data shown has been corrected for natural abundance. The MIDs were combined with ^13^C glucose data to simulate fluxes in Table S5.

**Table S6. Calculated fluxes for shPDHA1-2 DOX H460 cells during exponential growth.** All units are umol/hr/mg protein. Exchange fluxes are scaled 0-100% with reference to citrate synthase. SSE=75.7 with expected SSE [54.6,103.2], DOF= 77.

| **Net Flux** | **Value** | **95% Confidence interval** | |
| --- | --- | --- | --- |
| **Glycolysis** |  |  |  |
| Glucose Uptake | 4.55 | [4.19, | 4.91] |
| HK | 4.55 | [4.19, | 4.91] |
| PGI | 4.55 | [4.19, | 4.91] |
| PFK | 4.55 | [4.19, | 4.91] |
| TPI | 4.55 | [4.19, | 4.91] |
| GAPDH | 9.09 | [8.38, | 9.8] |
| ENO | 9.05 | [8.34, | 9.8] |
| PK | 9.05 | [8.34, | 9.76] |
| LDH | 9.03 | [8.31, | 9.74] |
| Lac Secretion | 9.03 | [8.31, | 9.74] |
| Pyr Transport | 0.4 | [0.35, | 0.45] |
| **TCA Cycle** |  |  |  |
| PDH | 0.14 | [0.11, | 0.17] |
| CS | 0.14 | [0.11, | 0.17] |
| ADH | 0.35 | [0.27, | 0.43] |
| SDH | 0.35 | [0.27, | 0.43] |
| FUM | 0.35 | [0.27, | 0.43] |
| MDH | 0.12 | [0.10, | 0.15] |
| **Anaplerosis/Cataplerosis** | |  |  |
| Gln Uptake | 0.81 | [0.69, | 0.93] |
| Gls | 0.78 | [0.67, | 0.90] |
| GDH | 0.44 | [0.34, | 0.55] |
| ME.m | 0.07 | [0.05, | 0.10] |
| ME.c | 0.38 | [0.27, | 0.49] |
| MDH.c | 0.23 | [0.18, | 0.28] |
| PC | 0.05 | [0.04, | 0.07] |
| PP | 0.04 | [0.03, | 0.05] |
| GOT2 | 0.04 | [0.03, | 0.05] |
| GPT | 0.28 | [0.26, | 0.31] |
| OC | 0.03 | [0.02, | 0.04] |
| Biomass | 0.0359 | [0.0277, | 0.0442] |

| **Citrate Modeling** |  |  |  |
| --- | --- | --- | --- |
| Cit Transport | 0.08 | [0.06, | 0.1] |
| Oxidative IDH | 0.06 | [0.05, | 0.07] |
| aKG Transport | -0.15 | [-0.19, | -0.12] |
| Reductive IDH | 0.15 | [0.12, | 0.19] |
| ACL | 0.23 | [0.18, | 0.28] |
| Internal Dilution | 4.8 | [2.6, | 6.9] |
| Cit.m -> Cit | 82.9 | [77.8, | 88.2] |
| Cit.c -> Cit | 12.3 | [7.4, | 17.1] |
| Cit-> Sink | 100 | [100, | 100] |

| **Exchange Flux** |  |  |  |
| --- | --- | --- | --- |
| LDH | 100 | [0, | 100] |
| TPI | 100 | [0, | 100] |
| GAPDH | 100 | [0, | 100] |
| ENO | 98.92 | [0.00, | 100] |
| SDH | 100 | [0.00, | 100] |
| FUM | 100.00 | [97.92, | 100] |
| MDH.m | 99.99 | [95.31, | 100] |
| GDH | 93.21 | [86.36, | 98.53] |
| MDH.C | 100 | [0.00, | 100] |
| GOT2 | 99.9 | [0.00, | 100] |
| **Additional Extracellular Fluxes** | |  |  |
| Glu Secretion | 0.3 | [0.26, | 0.37] |
| Ala Secretion | 0.23 | [0.21, | 0.25] |
| Asp Secretion | 0.007 | [0.006, | 0.008] |
| **Compartmentalization/Mixing** | |  |  |
| Mal.m -> Mal | 100 | [0, | 100] |
| Mal.c -> Mal | 0 | [0, | 100] |
| Mal -> Sink | 100 | [100, | 100] |
| Mal.m <-> Mal.c (Net) | 0.15 | [0.08, | 0.23] |
| Mal.m <-> Mal.c (Exchange) | 100 | [83.72, | 100] |
| Asp.m -> Asp | 100 | [0, | 100] |
| Asp.c -> Asp | 0 | [0, | 100] |
| Asp -> Sink | 100 | [100, | 100] |
| Asp.m <-> Asp.c (Net) | 0.04 | [0.03, | 0.05] |
| Asp.m <-> Asp.c (Exchange) | 100 | [0, | 100] |

**
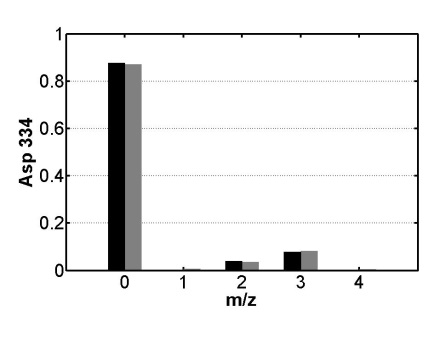

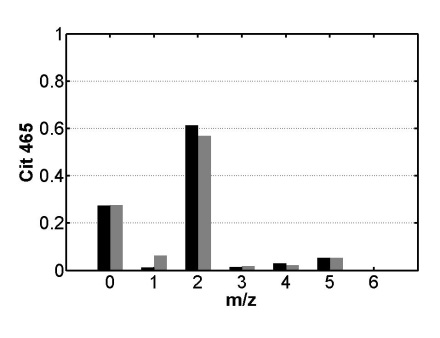

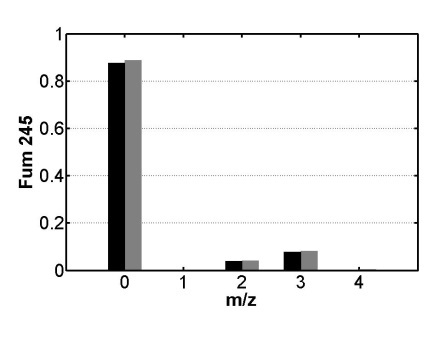

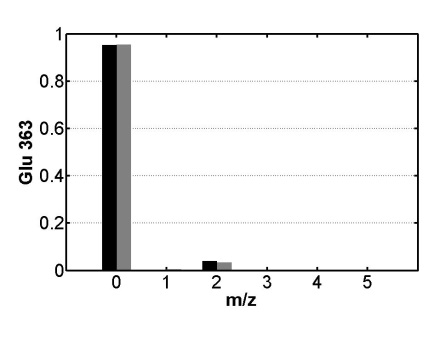

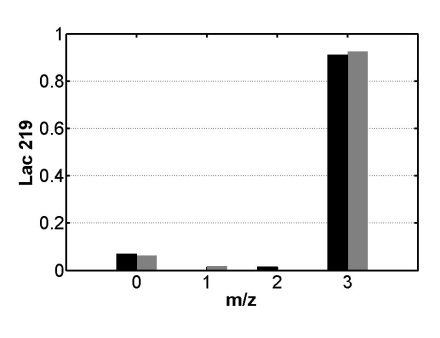

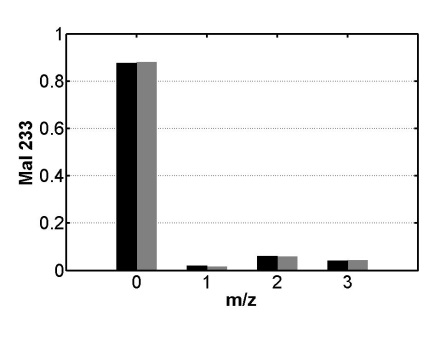

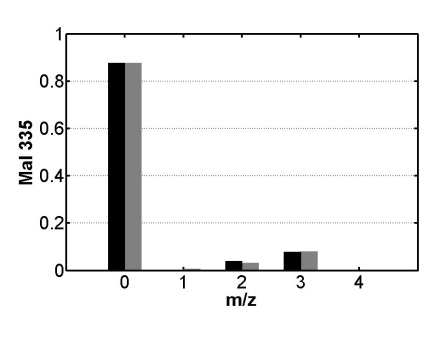

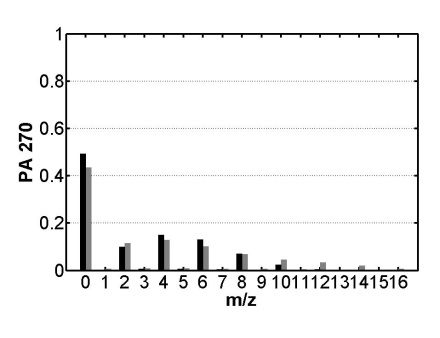

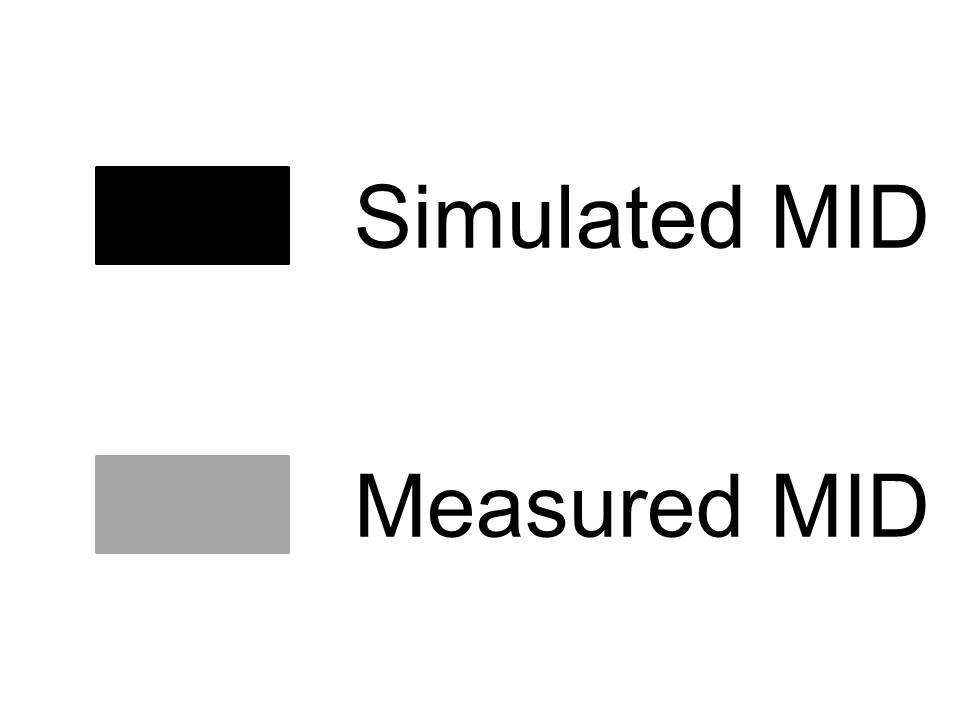
**

**Figure S11. Simulated and measured MIDs for shPDHA1-2 DOX H460 cells fed ^13^C glucose.** The data shown has been corrected for natural abundance. The MIDs were combined with ^13^C glutamine data to simulate fluxes in Table S6.

**
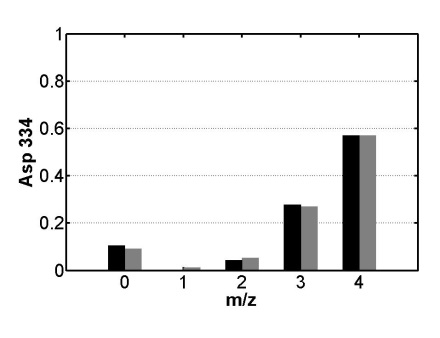

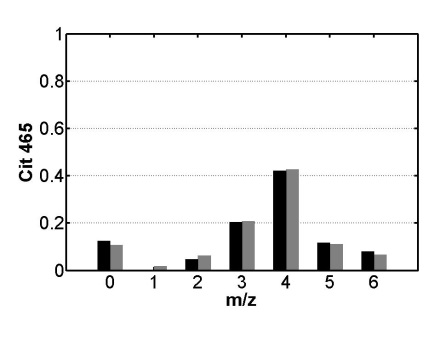

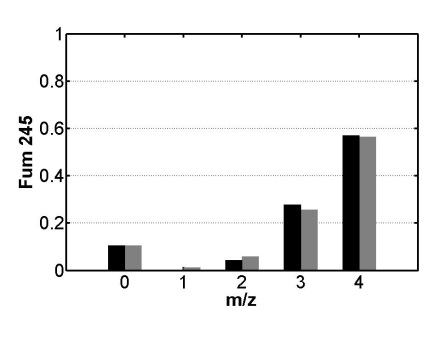

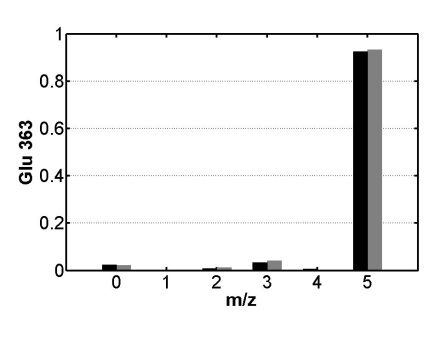

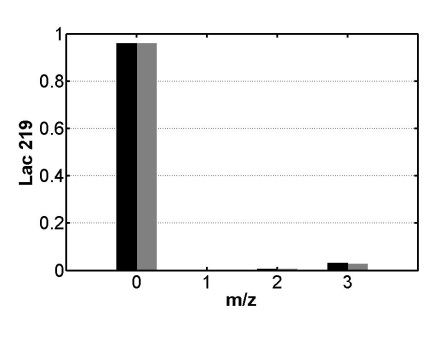

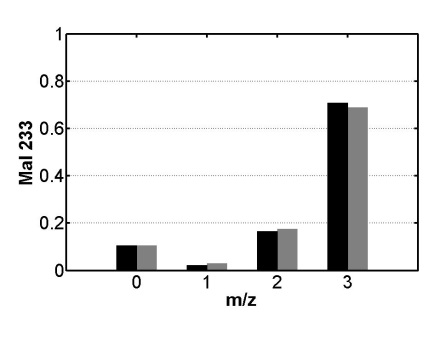

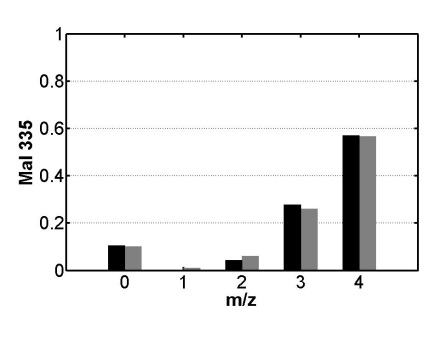

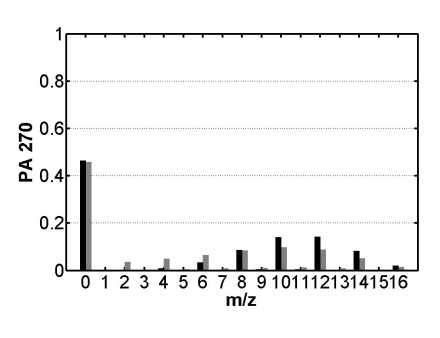

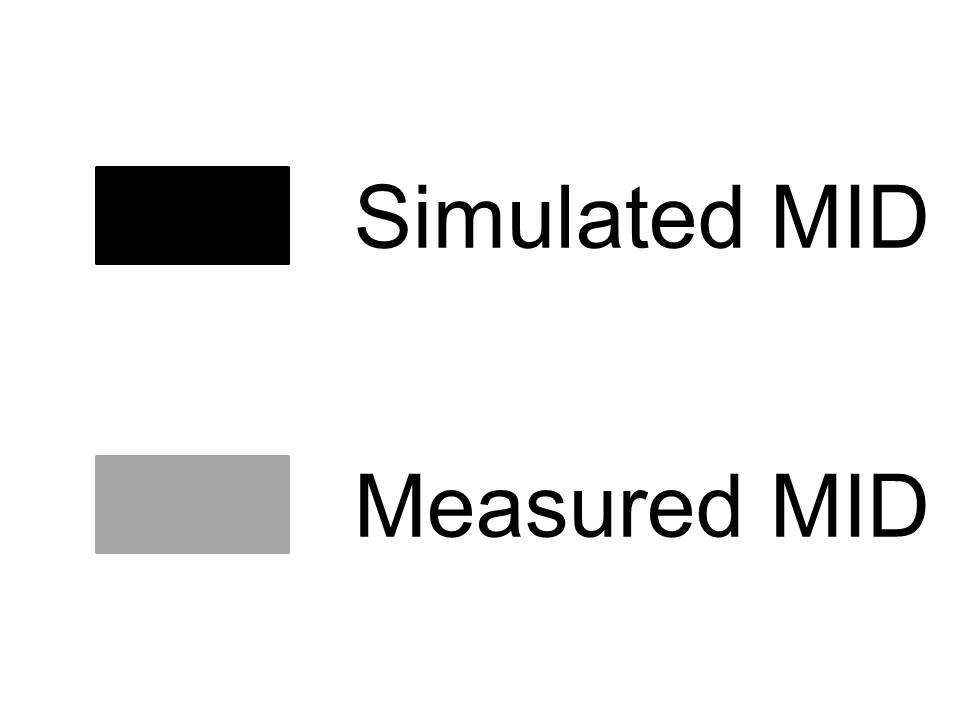
**

**Figure S12. Simulated and measured MIDs for shPDHA1-2 DOX H460 cells fed ^13^C glutamine.** The data shown has been corrected for natural abundance. The MIDs were combined with ^13^C glucose data to simulate fluxes in Table S6.

**References**

1. Ahn WS, Antoniewicz MR. Parallel labeling experiments with [1,2-(13)C]glucose and [U-(13)C]glutamine provide new insights into CHO cell metabolism. Metabolic engineering. 2013;15:34-47. doi:10.1016/j.ymben.2012.10.001.

2. Murphy TA, Dang CV, Young JD. Isotopically nonstationary 13C flux analysis of Myc-induced metabolic reprogramming in B-cells. Metabolic engineering. 2013;15:206-17. doi:10.1016/j.ymben.2012.07.008.

3. Templeton N, Dean J, Reddy P, Young JD. Peak antibody production is associated with increased oxidative metabolism in an industrially relevant fed-batch CHO cell culture. Biotechnology and bioengineering. 2013;110(7):2013-24. doi:10.1002/bit.24858.

4. Wiechert W, Siefke C, de Graaf AA, Marx A. Bidirectional reaction steps in metabolic networks: II. Flux estimation and statistical analysis. Biotechnology and bioengineering. 1997;55(1):118-35. doi:10.1002/(SICI)1097-0290(19970705)55:1<118::AID-BIT13>3.0.CO;2-I.
